# Epigenetic reader complexes of the human malaria parasite, *Plasmodium falciparum*

**DOI:** 10.1093/nar/gkz1044

**Published:** 2019-11-15

**Authors:** Wieteke Anna Maria Hoeijmakers, Jun Miao, Sabine Schmidt, Christa Geeke Toenhake, Sony Shrestha, Jeron Venhuizen, Rob Henderson, Jakob Birnbaum, Sonja Ghidelli-Disse, Gerard Drewes, Liwang Cui, Hendrik Gerard Stunnenberg, Tobias Spielmann, Richárd Bártfai

**Affiliations:** 1 Department of Molecular Biology, Radboud University, Nijmegen 6525 GA, the Netherlands; 2 Department of Internal Medicine, Morsani College of Medicine, University of South Florida, Tampa, FL 33612, USA; 3 Department of Entomology, Pennsylvania State University, University Park, PA 16802, USA; 4 Molecular Biology and Immunology Section, Bernhard Nocht Institute for Tropical Medicine, Hamburg D-20359, Germany; 5 TropIQ Health Sciences, Nijmegen 6534 AT, the Netherlands; 6 Cellzome GmbH, a GlaxoSmithKline Company, Heidelberg 69117, Germany; 7 Princess Maxima Center for Pediatric Oncology, Utrecht 3584CS, the Netherlands

## Abstract

Epigenetic regulatory mechanisms are central to the development and survival of all eukaryotic organisms. These mechanisms critically depend on the marking of chromatin domains with distinctive histone tail modifications (PTMs) and their recognition by effector protein complexes. Here we used quantitative proteomic approaches to unveil interactions between PTMs and associated reader protein complexes of *Plasmodium falciparum*, a unicellular parasite causing malaria. Histone peptide pull-downs with the most prominent and/or parasite-specific PTMs revealed the binding preference for 14 putative and novel reader proteins. Amongst others, they highlighted the acetylation-level-dependent recruitment of the BDP1/BDP2 complex and identified an PhD-finger protein (PHD 1, PF3D7_1008100) that could mediate a cross-talk between H3K4me2/3 and H3K9ac marks. Tagging and interaction proteomics of 12 identified proteins unveiled the composition of 5 major epigenetic complexes, including the elusive TBP-associated-factor complex as well as two distinct GCN5/ADA2 complexes. Furthermore, it has highlighted a remarkable degree of interaction between these five (sub)complexes. Collectively, this study provides an extensive inventory of PTM-reader interactions and composition of epigenetic complexes. It will not only fuel further explorations of gene regulation amongst ancient eukaryotes, but also provides a stepping stone for exploration of PTM-reader interactions for antimalarial drug development.

## INTRODUCTION

Malaria is caused by unicellular, eukaryotic parasites from the *Plasmodium* genus. *Plasmodium falciparum* parasites have an intricate lifecycle between their mosquito vector and human host, where all disease symptoms are associated with the asexual replication of the parasites within red blood cells (1). Growing evidence indicates the key role for chromatin-based, epigenetic regulatory mechanisms in governing life-cycle progression and enabling adaptation of the parasite (reviewed in (2)). The parasites possess a sizable collection of putative histone modifying enzymes (3,4). Many of these enzymes are essential for intraerythrocytic development (5) and are considered as potential drug targets (6–8). In addition, pioneering proteomic analysis of histone extracts identified more than forty posttranslational histone modifications (PTMs) and four different histone variants (H2A.Z, H2B.Z, H3.3, CenH3; (9,10), while more recent studies suggest that the number of PTMs and their combinations could easily exceed a hundred (11,12). These PTMs include methylation, acetylation, phosphorylation, ubiquitinoylation, formylation, crotonylation, amongst which acetyl modifications are the most numerous and abundant epigenetic marks (9). Genome-wide mapping of some of these PTMs and histone variants revealed the basic layout of the *Plasmodium* epigenome and revealed the key role for histone variants/modifications in dividing the genome into functionally distinct domains (reviewed in (13). H3K9me3/HP1-mediated heterochromatin formation at the chromosome ends and some chromosome internal islands (14–16), amongst others, contributes to antigenic variation, altered drug sensitivity and controls gametocyte production and hence the rate of transmission (17–19). The larger part of the epigenome on the other hand is in a transcriptionally permissive, euchromatic state. In this domain, intergenic regions are demarcated by a parasite-specific double-variant nucleosome (containing H2A.Z and H2B.Z; (20,21) and dynamically marked by several ‘activating’ histone modifications, including H3K4me3 and H3K9ac (20,22,23). Interestingly, the level of acetylation on some of these residues (e.g. H3K9 and H4) displays clear correlation with the transcriptional activity of the downstream gene (20,23).

While these studies highlighted the general organisation of the epigenome and revealed association between some histone modifications and gene activation/silencing, it is still not understood how these PTMs are interpreted by the parasite and how epigenetic reader, writer and eraser proteins work together to orchestrate the observed gene expression changes and enable survival of the parasite. While the *Plasmodium* genome encodes for nearly 30 putative reader proteins that could recognise these histone modifications (Supplementary Table S1), to date only a few of those have been characterized to some extent. For example, a bromodomain protein 1 (BDP1) has been shown to bind to acetyl modifications and interact with another bromodomain protein (BDP2) and with the transcription factor AP2-I to control expression of invasion related genes (24–26). Pf14-3-3-I binds to phosphorylated H3S28 (27), while SET10 is a histone methyltransferase with a PHD-domain implicated in the maintenance of the mutually exclusive expression of the active antigenic variation gene (28). Recently, a potent chemical probe, L-45 that binds with high selectivity to the PCAF and GCN5 bromodomain has been shown to co-crystallize with the bromodomain of *Pf*GCN5, providing an important proof of principle for the drugability of reader proteins in the malaria parasite (29).

Hence, in this study we set out to identify histone PTM-reader interactions on a large scale, as well as gaining insight in the ensemble of proteins that reside in these epigenetic ‘reader-complexes’. Hereby, we aim to obtain insights that could provide novel targets for drug-based intervention strategies.

## MATERIALS AND METHODS

### Detailed protocols are available in supplemental materials and methods.

#### Parasite culturing

Blood-stage *P. falciparum* parasites were maintained in a shaking, semi-automated 37°C incubator under low oxygen conditions (gas composition 3% O_2_, 4% CO_2_ and 93% N_2_) in human O+ red blood cells at 5% hematocrit in standard RPMI medium supplemented with 10% human serum or 0.5% Albumax (Life Technologies) and 0.2% NaHCO_3_. Wild-type 3D7 parasites were grown in the absence of antibiotics, while integrated transgenic lines (see below) were maintained in the presence of 400 μg/ml Geneticin G-418 Sulpate (Geneticin Selective Antibiotic (G418 Sulpate), Gibco, Thermo Fisher Scientific, cat# 11811031, dissolved to 50 mg/ml in MQ). Growth characteristics of the TAF1/BDP5 knock-sideways parasites were tested by FACS analysis and morphological investigation of parasites grown in the presence and absence of 250 nM rapalog (as in (30), Supplemental Materials and Methods).

#### Plasmid DNA cloning

For IP-MS/MS experiments, endogenous proteins were C-terminally tagged with a GFP or triple-HA tag using the selection-linked integration (SLI) system (30). In addition to a GFP- or HA-‘fishing’ moiety, plasmids were constructed to include two options for conditional knock-down to allow optimal flexibility. The self-cleaving GlmS ribozyme sequence for degradation of the mRNA (for both GFP- and 3xHA-tagged proteins) (31) and the auxin-inducible degron (AID) system for knockdown at the protein level (only for GFP-tagged proteins) (32). For two proteins, PF3D7_1212900/BDP2 and PF3D7_1008100/PHD1 3xHA epitope tag was used. For detailed cloning steps, refer to the Supplemental Materials and Methods. ‘GFP tagged’ line for PF3D7_1451200 was previously published (30).

The sequence encoding the 292 terminal amino acids of BDP5 was PCR amplified using primers BDP5for and BDP5rev (Supplementary Table S5) and cloned into pSLI-sandwich plasmid (30) using NotI and AvrII. For SLI-TGD, base pairs 4–801 of the *bdp5* gene were PCR amplified with primers BDP5-TGDfor and BDP5-TGDrev (Supplementary Table S5) and cloned into pSLI-TGD (30) using Gibson cloning. The correct sequence of cloned inserts was verified by sequencing.

#### Parasite transfection and generation of integrated lines

3D7 wt parasites were transfected following the procedure of Fidock and Wellems (33) using a BTX electroporation system. 12–24 h after transfection, selection for episomal transfectants was performed by addition of 2.6 nM WR99210 (Jacobus Pharmaceutical Company, Inc.). Parasites were cultured in the presence of WR99210 until they reached >10% parasitemia. Subsequently, selection for integrated parasites was started by addition of 400 μg/ml Geneticin G-418 sulphate. For transfection of *P. falciparum* parasites in the Spielman lab, 100 μg of purified plasmid DNA (QIAGEN) were transfected with an Amaxa system (Nucleofector II AAD-1001N, program U-033) as previously described (34). For details on validation of the correct integration of the constructs please refer to Supplemental Materials and Methods.

#### Quantitative histone peptide pulldown

Mixed stage, asynchronous *P. falciparum* parasites were grown for nuclear collection as described in ‘*Plasmodium falciparum* culturing’. The nuclear collection protocol was modified from (20). Nuclear extract was generated as in (35).

The histone peptide pulldown protocol was modified from (36,37) and protocols made available by Cellzome (for details see Supplemental Materials and Methods). Most experiments were designed to contain three dimethyl labels (‘light’, ‘medium’, ‘heavy’) and included two modified histone peptide reactions and a common unmodified control peptide reaction for quantitative analysis. The unmodified control peptide was of the exact same sequence as the modified peptide(s), but lacked the specified PTMs. Peptides are listed in Supplemental Materials and Methods. Technical replicates were included for each nuclear extract with subsequent numbering (e.g. experiments 1 and 2 are always technical replicates) and were performed using label-swap conditions for dimethyl labelling (uneven numbered experiments were performed as forward reactions (i.e. experimental pull-down is heavy isotope labelled, while control is light isotope labelled); even numbered experiments were reverse reactions (i.e. experimental pull-down is light isotope labelled, while control is heavy isotope labelled)). For each peptide, except H3_2ac (K9acK14ac), multiple biological replicates were performed from independent nuclear extracts.

#### Recombinant PHD1 PHD-domain peptide binding assay

The fourth PfPHD1 PHD domain was expressed in *Escherichia coli* as a GST-fusion protein. Briefly, a gene fragment coding the fourth PHD domain of *Pf*PHD1 (nucleotides 11374–11562, amino acids 3792–3853) was cloned into pGEX-6P-1 vector. Soluble GST-fusion protein was purified by glutathione sepharose 4b (GE Healthcare) based on the manufacture's protocol. The peptide binding assay was conducted as described earlier by Chang and colleagues (38).

#### Quantitative GFP- and HA-pulldowns

The GFP- and HA-pulldown procedures were modified from (39). Experiments were set up as technical replicates, which were numbered sequentially (e.g. forward reactions contain uneven numbers, reverse reactions are even numbered). Biological replicate experiments from independent nuclear extract were also performed for each bait. For each experiment a GFP- or HA-binding reaction was performed in parallel to a negative control pulldown on the same input material. Technical replicates were performed under label-swap conditions using ‘light’ and ‘heavy’ dimethyl labelling (as in the HPP experiments above). Quantitative proteomics was performed to distinguish proteins binding significantly to the GFP- or HA-beads over background proteins. In order to exclude false-positive proteins that are not part of the tagged-proteins complex, but show direct enhanced binding to the GFP-Trap of HA-antibody beads, two negative controls were included. (i) a HA-negative control in which HA-bead and control bead pulldowns were performed from a nuclear extract from GFP-tagged parasites; and (ii) a GFP-negative control in which GFP-Trap and control bead pulldowns were performed on a wild-type (experiments 3 and 4) or HA-tagged (experiments 1 and 2) nuclear extract. For further details see Supplemental Materials and Methods.

#### Microscopy

Fluorescence microscopy was carried out essentially as described (40). Briefly, 1 μg/μl DAPI was added to the parasites in culture medium and incubated for 15 min at room temperature. For imaging, a drop of this suspension was placed on a glass slide and covered with a coverslip. Images were acquired with a Zeiss AxioImager M1 or M2 microscope with a Hamamatsu Orca C4742–95 camera controlled by AxioVision software. A 63× plan-apochromate oil immersion objectives with an aperture of 1.4 was used for all images. Brightness and intensity of images adjustments and overlays were done using Corel Photo Paint (version X6). To assess knock sideways efficiency, a line crossing the nucleus was drawn through fluorescence images. The ‘plot profile’ function of ImageJ was then used to assess the fluorescence intensity of the DAPI signal and this was compared with the intensity profile of the same line in the GFP (TAF1/BDP5) signal.

#### Comparative transcriptome analysis (RNA-sequencing)

TAF1/BDP5–2xFKBP-GFP-2xFKBP parasites with and without the Lyn-FRB-T2A-mCherry mislocalizer plasmid (30) were synchronized to a ∼4 h time window. At 14 h post invasion, cultures were split and one half was treated with 250 nM rapalog. Parasites with the mislocalizer were collected at 2, 6 and 12 h after knock sideways induction. Control parasites without the mislocalizer were collected after 12 h of rapalog treatment. Total RNA was isolated using the Qiagen RNeasy kit and transcripts with polyA tail were purified using the Oligotex mRNA Mini Kit (Qiagen, #70022). RNA fragmentation, cDNA synthesis and directional RNA-seq library generation were performed as described in (35). Libraries were sequenced on a NextSeq 500 system (Illumina) for 2 × 42 bp. Reads were mapped against the *P. falciparum* 3D7 Transcriptome (PlasmoDB v26) using BWA resulting in 14.1–22.9M mapped reads per library. Reads mapping to the sense strand of the transcripts were normalized using the reads-per-million-per-kilobase (RPKM) method. Differentially expressed genes with a minimum 2-fold change in mRNA abundance (up or down) in the rapalog condition compared to the control with a minimum RPKM of 5 in either in the control or treated sample were further analyzed. Relative or absolute expression of DE genes were plotted at different RBC, gametocyte and ookinete stages using data from (26) and (41).

#### Mass spectrometry

For each Histone Peptide Pulldown sample pool, a single 3xC18 disk stage-tip was eluted. For each GFP- or HA-pulldown sample pool either one or two 5xC18-disk stage-tips were eluted. Stage-tips that were loaded directly before elution omitted the rehydration step. Peptides were eluted using 30 μl buffer B (80% acetonitrile, 0.1% formic acid) per stage-tip in PCR-tubes. Acetonitrile evaporation was achieved by 15–35 min vacuum spin at room temperature to ∼5–8 μl volume, after which each sample was reconstituted to 12 μl with buffer A. 10 μl of reconstituted sample was separated over a 30 cm C18-reverse phase column (1.8 μm Reprosil-Pur C18-AQ, Dr Maisch 9852) and eluted using an Easy-nLC 1000 (Thermo Fisher Scientific) over a 94 min gradient (5.6% acetonitrile/0.1% formic acid—25.6% acetonitrile/0.1% formic acid). Eluted peptides were directly injected into a QExactive mass spectrometer (Thermo Scientific). Data was acquired in TOP10 data-dependent acquisition mode with dynamic exclusion enabled for 20 s. Resolution for MS was set at 70 000 at *m*/*z* = 400 and for MS/MS at 175 000.

#### MS data processing

Raw mass spectra were analyzed similar to (35). All histone peptide pulldown MS raw files were combined in a single analysis run to increase protein identification and comparability between individual pulldowns. For the same reason all GFP- and HA-pulldown MS raw files that are displayed together in a single figure were combined into one analysis. Xcalibur raw files were processed using MaxQuant (version 1.5.3.30 (42)) set to default parameters unless indicated. Match-between-runs and re-quantify options were enabled with default parameters and iBAQ values were calculated. Mass spectra were compared to peptide masses from the *P. falciparum* 3D7 annotated proteome (PlasmoDB release 33) with the entire human proteome included in the contaminants list using the integrated Andromeda search engine. Default search settings (mass tolerance at 4.5 ppm for precursor ions and 20 ppm for fragment ions) were enabled, and peptides and proteins were accepted with an 0.01 FDR cut-off. Protein quantification required minimally a single ‘unique + razor’ peptide-ratio to increase assay sensitivity. However, since single-peptide identifications are more prone to variation, more stringent filtering for at least two peptides has been employed in most downstream analysis (as indicated).

The MaxQuant output ProteinGroup file was further analyzed using the Perseus software package (version 1.4.0.20 (43)). Further processing of the Histone Peptide pulldown and GFP-/HA-pulldown files was performed in excel. For specific details on the HPP and GFP/HA-pull-down analysis please refer to the Supplemental Material and Methods.

## RESULTS

### Histone peptide pulldown enables identification of histone readers and associated proteins

**Figure 1.**
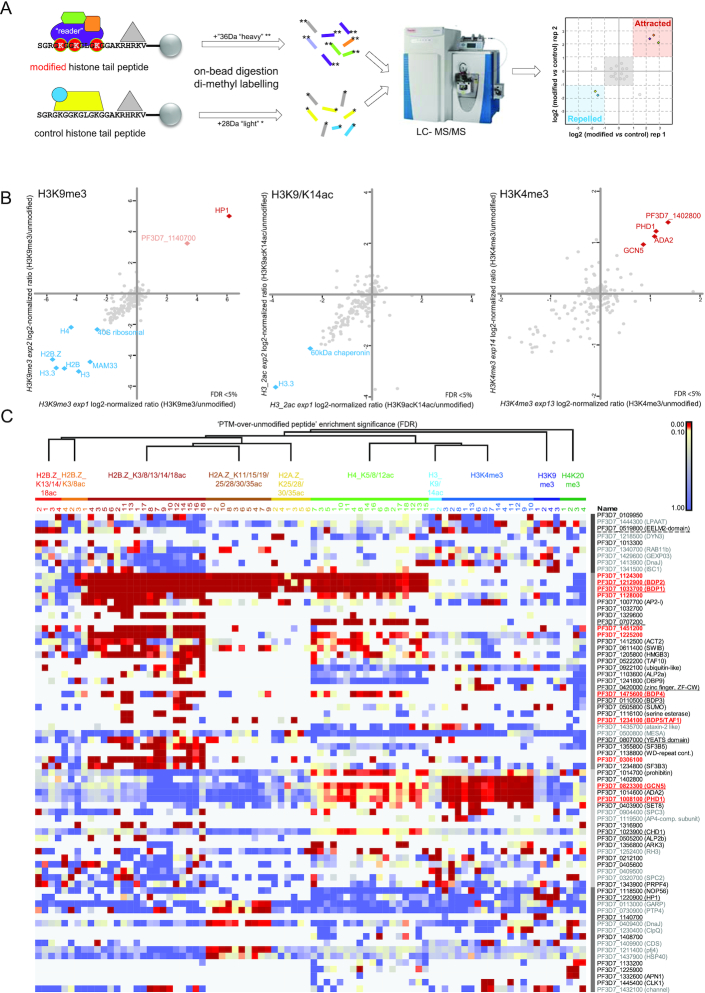
Histone peptide pulldown enables identification of histone readers and associated proteins. (**A**) Schematic overview of the histone peptide pull-down method. Peptide corresponding to a specific histone tail modification as well as unmodified control peptide are incubated with nuclear extract to enrich for reader proteins and associated protein complexes. These proteins are then subjected to on bead trypsin digestion followed by dimethyl labelling of the resulting peptides. Finally, light and heavy labeled peptides are mixed and simultaneously analyzed on a LC-MS/MS machine to define the relative abundance of the corresponding proteins. (**B**) Scatterplots of log_2_ normalized ratios of protein enrichments (modified/unmodified) in histone peptide pull downs using H3K9me3, K3K9/14ac and H3K4me3 as well as unmodified control peptides. Proteins significantly enriched/repelled (FDR < 5%) in both experiments are highlighted with red or blue diamonds, respectively. For scatterplots displaying all ‘repelled’ proteins see Supplementary Figure S1. Notably, the protein PF3D7_1140700 was manually added to H3K9me3 scatterplot (for explanation, see text). (**C**) Heat map displaying the false discovery rate (FDR) for all histone peptide pulldown experiments. Only proteins significantly enriched with FDR <5% in at least 20% of the reactions for each PTM are included. Columns and rows were ordered using sequential hierarchical clustering. Clusters are indicated in blocks with different shades of grey in between heatmap and protein IDs. Red proteins were selected for tagging and used in IP-MS/MS experiments (Figure 4). Underlined proteins contain putative reader domain (solid line) or represent putative novel reader proteins (dashed line). Proteins that are less likely to be real interactors of PTMs or that of reader proteins based on their marginal enrichment, proven non-nuclear localization or predicted function are listed in grey.

In order to obtain insight into the protein machinery interpreting epigenomic marks, we aimed to identify the reader proteins recruited to specific histone modifications in *Plasmodium*. Therefore, we adapted histone peptide pulldown technology (HPP; (44)) to *P. falciparum*. We used synthetic histone tail peptides carrying one or more specific epigenetic modification(s) to enrich for proteins binding to these histone marks from nuclear extract of mixed-stage intraerythrocytic *P. falciparum* parasites. Proteins preferentially interacting with the modified peptide as compared to the unmodified control peptide were identified by a quantitative proteomic workflow using dimethyl labelling and simultaneous quantification of tryptic peptides (Figure 1A). We tested this approach on three conserved epigenetic marks of which the genome-wide localization during the Plasmodium asexual cycle has been defined (15,20): H3K9me3, H3K9acK14ac and H3K4me3. H3K9me3 served as our positive control, given that its interaction with heterochromatin protein 1 is well established (15,45). Indeed, we found HP1 strongly enriched in our pulldown experiments (Figure 1B). Interestingly, next to HP1 we also identified another chromodomain-containing protein (PF3D7_1140700) in our H3K9me3 pull-downs. Although this protein was only detected in two out of four reactions (Figure 1C, manually added to Figure 1B), the presence of a reader-domain that could mediate a direct interaction with the recruiting methyl mark, make this an interesting candidate for further exploration. While H3K9/14ac has been suggested to be recognised by bromodomain protein 1 (BDP1; (24) we did not detect this interaction perhaps due to the substantially higher detergent concentration used in our experiments (Figure 1B). The third mark used to test the *P. falciparum* histone peptide pulldown, revealed several proteins specifically recruited to H3K4me3: GCN5, ADA2, a zinc finger protein (PF3D7_1008100) and a protein with unknown function (PF3D7_1402800). Since only one of these proteins (PF3D7_1008100), has predicted methyl-lysine binding activity (PhD-finger domain, hence we named it PHD1) and GCN5 and ADA2 are conserved interactors (46,47), we reasoned that these proteins might form a complex that is specifically recruited to this modification (discussed in detail later).

Notably, we also identified several proteins significantly enriched by the unmodified peptide. These could, in theory, be reader proteins specifically binding to unmodified histone tails and repelled by the tested modifications (Figure 1B, auto-scaled plots in Supplementary Figure S1). However, these ‘repelled’ proteins almost always consisted of highly abundant histones, chaperones or ribosomal proteins, which are common ‘false positives’ in proteomic experiments. Hence, they are unlikely to be relevant interactors of unmodified histone tails.

Collectively, these experiments established HPP as a valid approach to identify known and novel epigenetic reader proteins as well as their associated proteins in *P. falciparum*.

### Readers of the acetylated and methylated histone tails of the *P. falciparum* epigenome

After demonstrating that histone peptide pulldown can be used to identify both reader proteins as well as their likely interactors in *P. falciparum*, we aimed to identify proteins and protein complexes recruited by other epigenetic marks. Of particular interest are acetyl marks on the parasite-specific tails of the histone variants, H2A.Z and H2B.Z. (9). To ensure that we investigate PTM combinations that occur *in vivo* and likely have some biological relevance, we selected specific combinations of 2–7 acetylated lysine residues that have been detected to co-occur on a single peptide in mass-spectrometry experiments by us (unpublished observations). These acetyl-combinations were also observed by others in *P. falciparum* (11) or in a related pathogen, *T. gondii* (48). In addition, we also included two prominent H4 modifications, which have been previously detected in *P. falciparum*; namely acetylation of lysines 5, 8 and 12 or trimethylation of lysine 20 (9,16,23).

Using 10 different combinations of histone PTMs and nuclear extracts collected from independent blood-stage parasite cultures we detected about fifty different proteins significantly (FDR < 5%) and consistently interacting with at least one of the epigenetic marks (Figure 1C, Supplementary Table S2). This list includes 12 bromo-, chromo-, YEAST- or PhD/CW-type zinc-finger-domain-containing proteins (underlined on Figure 1C), comprising about half of the known or putative reader proteins (Supplementary Table S1). *K*-means clustering of proteins significantly enriched for the modified peptides highlighted putative epigenetic protein complexes (Figure 1C). Among these, a complex consisting of two bromo-domain proteins (BDP1 and 2) and a protein with unknown function (PF3D7_1124300) was recruited to nearly all acetylated peptides (discussed in detail in the next paragraph). Of interest, the transcription factor AP2-I was previously shown to be associated with BDP1 and BDP2 (25,26). In line with this finding, we observe a clear recruitment of BDP1, BDP2 and AP2-I by the H2B.Z_K3/8/13/14/18ac peptide. In contrast, however, we find at best low levels of recruitment of AP2-I by the H4K5/8/12ac peptide, while BDP1, BDP2 and PF3D7_1124300 are very strongly recruited. This suggests that while the ‘BDP1/BDP2-core complex’ is recruited to many different acetyl modifications, the presence of additional components might differ per epigenetic mark. The existence of such alternative BDP1 complexes has earlier been postulated by Santos *et al.* (25).

Similarly, next to H3K4me3 (see above) the GCN5/ADA2 complex was also recruited to H4K5/8/12ac, although to a much lesser extent. This is most likely mediated through a direct interaction with the bromo-domain of GCN5. Interestingly, however, a putative protein component of this complex without known function (PF3D7_1402800) is consistently co-recruited in the context of H3K4me3 binding, but not when H4K5/8/12ac peptides are used (Figure 1C), again suggesting the existence of epigenetic mark specific complex composition.

**Figure 2.**
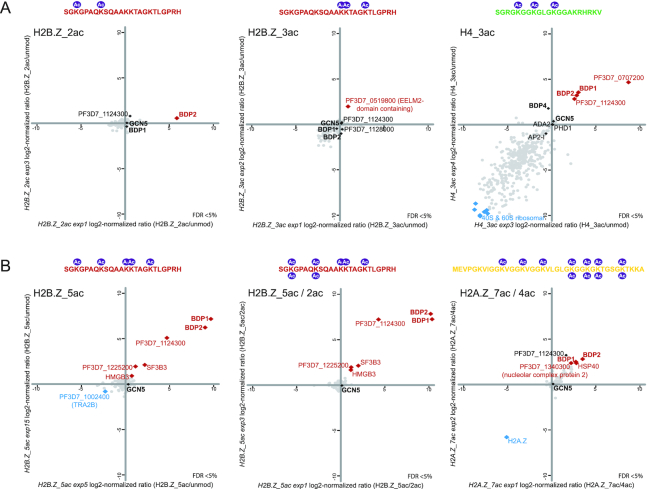
Bromodomain readers exhibit moderate site-specificity and are recruited in an acetylation level-dependent manner. (**A**) Scatterplots of log2 normalized ratios of protein enrichments in histone peptide pull downs for H2B.Z_2ac (H2B.Z_K3/8ac), H2B.Z_3ac (H2B.Z_13/14/18ac) and H4_3ac (H4K5/8/12ac) over unmodified control peptides. Scatterplots of the same experiments with auto-scaled axes are shown in Supplementary Figure S2. Histone peptide sequences and modifications are depicted above the scatterplot. (**B**) Scatterplots of log_2_ normalized ratios of protein enrichments in histone peptide pull downs: H2B.Z_5ac (H2B.Z_K3/8/13/14/18ac) over unmodified; H2B.Z_5ac over 2ac as well as H2A.Z_7ac over 4ac peptide. Scatterplots of the same experiments with auto-scaled axes are shown in Supplementary Figure S2. Histone peptide sequences and modifications are depicted above the scatterplot.

Notably, many other proteins without recognisable reader domain were recruited to specific histone marks. An interesting possibility is that some of these proteins might represent novel types of reader proteins. For example, PF3D7_0707200 is very specifically and prominently recruited to H4K5/8/12ac (Figure 1C, Figure 2A), but does not contain any recognisable reader domain(s). Similarly, an EELM2-domain-containing protein (PF3D7_0519800), without any known reader domains, is the only protein consistently recruited to H2B.Z_K13/14/18ac (Figures 1C, 2A; (49)).

Collectively, these HPPs revealed interactions between several histone modifications and putative or novel reader proteins as well as their likely interactors and suggest the existence of modification-specific ‘flavours’ of reader protein complexes.

### Bromodomain reader complexes exhibit moderate site-specificity and are recruited in an acetylation level-dependent manner

Interestingly, most bromodomain proteins (except for GCN5) are recruited to more than one acetylated peptide and in particular to highly acetylated histone tails (Figure 1C). Importantly, however, this does not mean a complete lack of selectivity. BDP5/TAF1, for example, is sporadically detected in H2B.Z_K3/8/13/14/18ac and H2A.Zac HPPs, but not in other pulldowns. Moreover, even the ‘BDP1/2 core complex’, which exhibits affinity to a broad range of acetyl modifications (H2B.ZK3/8ac, H2B.Z_K3/8/13/14/18ac, H2A.Z_K25/28/30/35ac & H2A.Z_K11/15/19/25/28/30/35ac & H4K5/8/12ac; Figure 1C, Figure 2) was not observed to bind to H2B.Z_K13/14/18ac (Figure 2A) and to H3K9/14ac peptides (Figure 1B).

Notably, the BDP1/BDP2 complex is recruited in an acetylation-level dependent-manner. While this complex is moderately enriched in H2B.ZK3/8ac HPPs (Figures 1C and 2A), it exhibits much stronger binding to the same peptide carrying penta-acetyl modification (H2B.Z_K3/8/13/14/18ac, Figure 2B). Similarly, we observed a slightly higher enrichment of BDP1 and BDP2 when ‘fishing’ with hepta-acetyl H2A.Z peptide as compared to its tetra-acetyl counterpart (Figure 2B). Collectively, these results suggest a rather loose sequence specificity of *P. falciparum* bromodomain protein complexes and overall an increased affinity towards highly acetylated histone tails.

### The HAT module of a SAGA-like complex is recruited to H3K4me2/me3 via an unconventional PhD-finger containing reader

**Figure 3.**
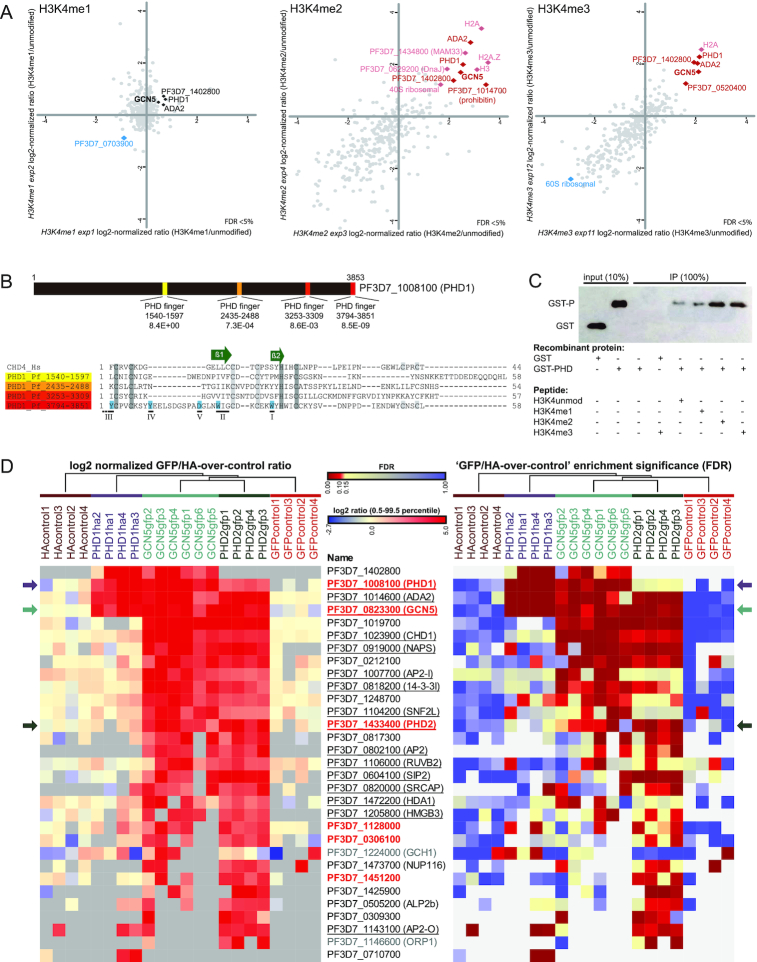
The HAT module of a SAGA-like complex is recruited to H3K4me2/me3 via an unconventional PhD-finger containing reader. (**A**) Scatterplots of log_2_ normalized ratios of protein enrichments in histone peptide pull downs: H3K4me1, me2 and me3 over unmodified peptide corresponding to the 21 amino acid N-terminal sequence of *P. falciparum* H3.3. Scatterplots with auto-scaled axes and heatmaps summarizing all H3K4me pulldown experiments can be found in Supplementary Figure S3A and B. Components of the GCN5/ADA2 core complex are highlighted (note that their enrichment on the H3K4me1 pull down is not significant). (**B**) The domain structure of PHD1 (PF3D7_1008100) as identified by the SMART algorithm (64) using default settings (top panel). Alignment of the PHD-domain sequences of PHD1 against human CHD4 PHD domain. Key features of PHD domains are highlighted on the alignment: conserved zinc-coordinating residues are labelled grey; two core β-strands are indicated by green arrows; regions involved in ligand recognition and selectivity are numbered I–V, with residues known to be important for H3K4me3 recognition highlighted blue (65). (**C**) Western blot quantifying the amount of recombinant glutathione S-transferase (GST) protein fused to the fourth PhD-finger of PHD1 before (10% input) or after pull-down with unmodified, K4me1, K4me2 or K4me3 H3.3 N-terminal peptides. The composition of each pull-down reaction is indicated below the image (+ present; - absent). (**D**) Heatmaps depicting the log2 normalized GFP/HA-over-control ratios as well as the false discovery rates (FDR) for proteins identified in co-immunoprecipitation experiments using parasite lines in which GCN5, PHD1 or PHD2 was endogenously tagged with GFP or 3xHA as indicated. Significant outliers were identified for each reaction by means of intensity-based outlier statistics (two-sided Benjamini–Hochberg test). Proteins significantly enriched with an FDR < 10% in at least two reactions for GCN5, PHD1 and/or PHD2 were selected, while excluding proteins significantly enriched with the same criteria in the GFP- and/or HA-negative controls using wild-type nuclear extracts. Columns were clustered using a sequential hierarchical clustering approach. Rows were ordered manually, first listing the proteins strongly recruited by PHD1 and then ranking based on the strength of recruitment in the combined GCN5 pulldown from high to low summed log_2_-norm ratios.

Our initial H3K4me3 pulldowns (Figure 1B), performed using a peptide corresponding to the 19 most N-terminal amino acids of *P. falciparum* histone H3.3, revealed recruitment of four proteins that could constitute part of a *P. falciparum* SAGA-like complex as they contained GCN5 and ADA2 (47). To further dissect the recruitment of GCN5/ADA2 to methylated H3K4 we performed HPPs with mono-, di- and tri-methylated peptides. Notably, these peptides were ordered from a different company and constituted 21 instead of 19 amino acids. The four proteins (GCN5, ADA2, PHD1, PF3D7_1402800) we earlier detected binding to H3K4me3 with the shorter peptide were consistently recruited to both H3K4me2 and H3K4me3, while they were not or very moderately recruited to H3K4me1 (Figure 3A, Supplementary Figure S3A). In addition, only with the longer H3K4me2/3 peptides we detected several other potential binders, including SET8, SSB, prohibitin, ARK3 and FACT-S (Figure 3A, Supplementary Figure S3A, B). Binding of these proteins might be enhanced by residues further from the modified lysine at position 4 or blocked by the linker module of the 19-aa peptide.

In contrast to the proteins above that show a variable or lower level of recruitment in H3K4me2/3 HPPs, recruitment of GCN5, ADA2, PHD1 and PF3D7_1402800 was very robust to the H3K4me2 and both lengths of H3K4me3 peptides. These proteins show a clear resemblance to the core of the HAT-module of the SAGA complex of other eukaryotes (47), where a GCN5-ADA2 is accompanied by the presence of a H3K4me3-reader. Interestingly, instead of a two tudor-domain-containing reader protein (SGF29), which is a conserved component of SAGA in many eukaryotes (47), the *P. falciparum* complex contains an unconventional putative reader protein with four PhD-finger domains (PF3D7_1008100, PHD1). After close examination of the amino acid sequence of the PHD domains, the fourth PhD domain of this protein was expected to most likely be responsible for recruitment to methylated H3K4 as it contains the critical residues known to mediate this interaction (Figure 3B; (50)). To confirm H3K4me2/3 binding experimentally, we recombinantly expressed this domain in fusion with glutathione-*S*-transferase (GST) and tested its capacity to bind to methylated or non-methylated H3-tail peptides *in vitro*. In agreement with the HPP data, this PhD-finger domain showed higher affinity to H3K4me2 and H3K4me3 than to H3K4me1 or the unmethylated control peptide (Figure 3C), demonstrating that this domain indeed can mediate the recruitment to H3K4me2/3.

To further characterize the composition of *P. falciparum* SAGA-like complex, we generated transgenic *P. falciparum* lines expressing GFP-tagged GCN5 or 3xHA-tagged PHD1 from the endogenous locus (Supplementary Figure S4). Immunoprecipitation of PHD1-HA confirmed GCN5, ADA2, PHD1 and PF3D7_1402800 as components of a core HAT module (Figure 3D, Supplementary Table S3), but did not identify additional SAGA-like complex components perhaps in part due to the lower sensitivity of the HA-IP. In contrast, the GCN5-GFP pulldown identified a much larger set of putative interacting proteins. These included another PhD-finger containing protein PF3D7_1433400 (which we named PHD2). The presence of PHD2 in addition to PHD1 in the GCN5-GFP pulldown triggered our attention given that a common concept of epigenetic complexes in other organisms is the existence of multiple flavours of a complex where functionally redundant proteins can substitute for each other in a mutually exclusive fashion. To test this hypothesis, we generated a transgenic *P. falciparum* line expressing endogenous PHD2 with a C-terminal GFP-tag (Supplementary Figure S4) and performed affinity purification followed by mass-spectrometry. Interestingly, while many of the interactors identified in the GCN5-GFP pulldown were clearly enriched in the PHD2 pulldown, PHD1 was only marginally enriched and PF3D7_1402800 was not identified (Figure 3D, Supplementary Figure S3C, Table S3). Although these data are no formal proof of the existence of multiple flavours of a SAGA-like complex, they hint to the existence of different flavours of this complex displaying different compositions and possibly distinct functions.

Collectively, these results demonstrate that a HAT-module, minimally containing GCN5, ADA2 and PF3D7_1402800, is recruited by a parasite-specific reader (PHD1) to H3K4me2/3. As GCN5 is known to acetylate H3K9/K14 in *P. falciparum* (51), our data is consistent with a crosstalk between these important epigenetic marks in the parasite. Moreover, our results are suggestive for the existence of another GCN5/ADA2 complex that instead of PHD1, contains PHD2, and is part of another epigenetic module (see next paragraph).

### Epigenetic-complexes of *P. falciparum* display remarkable connectivity

**Figure 4.**
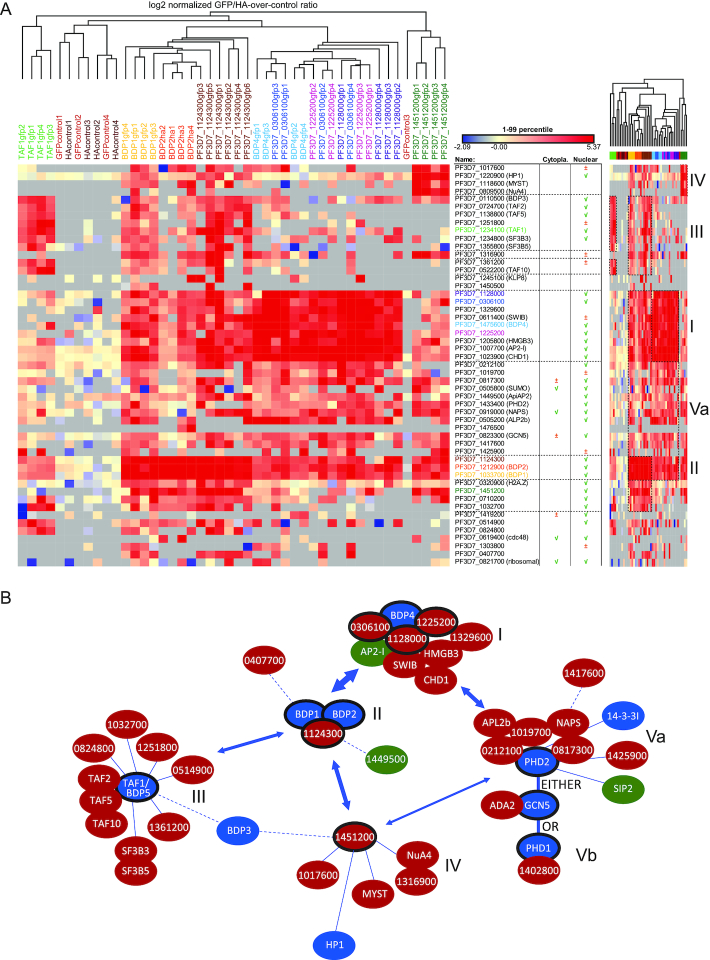
Epigenetic-complexes of *P. falciparum* display remarkable connectivity. (**A**) Heatmap depicting the log2 normalized GFP/HA-over-control ratios for proteins identified in co-immunoprecipitation experiments using nuclear extract from parasite lines in which either PF3D7_0306100; PF3D7_1033700/BDP1; PF3D7_1124300; PF3D7_1128000; PF3D7_1225200; PF3D7_1234100/TAF1-BDP5; PF3D7_1451200; PF3D7_1475600/BDP4; or PF3D7_1212900/BDP2, respectively, were endogenously tagged with GFP or 3xHA as indicated. Significant outliers were identified for each reaction by means of intensity-base outlier statistics (two-sided Benjamini-Hochberg test). Proteins significantly enriched with an FDR < 10% in at least 2 IP reactions were selected, while excluding proteins significantly enriched with the same criteria in the GFP- and/or HA-negative controls using wild-type nuclear extracts. Columns and rows were clustered using a hierarchical clustering. Previous detection of proteins in cytoplasm and/or nucleus is included based on the dataset of (49). A compressed heatmap is included on the right side of the figure where specific clusters of proteins are highlighted and numbered: I) BDP4-complex; II) BDP1/2/PF3D7_1124300-core complex; III) TAF1/BDP5-complex; IV) PF3D7_1451200-specific interactors and Va) PHD2/GCN5/ADA2-complex. (**B**) A model of *P. falciparum* epigenetic-complex compositions and connectivity. All nodes were manually positioned in the network plot based on evidence from: (i) our immunoprecipitation experiments (Figure 3D and Figure 4A); (ii) our histone peptide pulldowns (Figure 1C); (iii) known interactions between orthologous proteins in other organisms, (iv) publicly available yeast-2-hybrid interaction data (66). Bait proteins used in IP experiments are encircled with a black line. Putative reader proteins are blue, while putative or known DNA-binding proteins are green. Complexes are numbered as in (A).

To dissect the composition of other epigenetic complexes, we endogenously epitope tagged and affinity purified nine other proteins that have been identified in our HPP experiments, mainly proteins with reader domains or proteins without known function (Supplementary Figure S4, Table S4). Clustering of the quantitative proteomic pull-down data revealed the composition of five main modules (i.e. groups of strongly associated proteins, Figure 4): BDP4 core complex (I); BDP1/2/1124300 core complex (II); TAF1(BDP5) complex (III); a group of loosely associated proteins consisting of PF3D7_1451200 interactors (IV) and the PHD2/SAGA-like complex described in detail in the previous paragraph (Va). Interestingly, our analysis highlighted a remarkable level of connectivity between these modules (Figure 4). For example, while BDP1/BDP2/PF3D7_1124300 as well as the BDP4 complex (BDP4, SWIB, CHD1, AP2-I, HMDB3, PF3D7_1128000, PF3D7_0306100, PF3D7_1329600 and PF3D7_1225200) represent two separate core modules, there is a very prominent interaction between these modules (Figure 4). Similarly, many members of these two modules also interact with the above described components of the PHD2/SAGA-like complex (NAPS, APL2b, PF3D7_0212100, PF3D7_1019700, PF3D7_0817300, PF3D7_1425900, but not PHD1 and PF3D7_1402800, see previous paragraph and Figures 3D, 4). Interestingly, TAF1/BDP5 co-precipitated with a distinct set of proteins and forms a separate module less closely connected to the other complexes (discussed in detail in the next paragraph).

Collectively, our GFP tagging followed by affinity purification mass spectrometry of 12 epigenetic complex components reveals the composition of five distinct epigenetic modules, which each might exist in multiple flavours. Intriguingly, we observed many interactions between these modules, highlighting a large degree of connectivity and a high level of complexity in epigenetic regulation of malaria parasites.

### TAF1/BDP5, a component of the elusive *P. falciparum* TAF module, is essential for asexual development

One of the bromodomain proteins (TAF1/BDP5) we analysed in IP-MS/MS revealed association with a distinct set of proteins. These proteins form a separate complex, which shows some association to the BDP1/2 core complex, but not to other modules (Figure 4). BDP5 itself was earlier postulated to be the TBP-associated factor 1 (TAF1) homologue of *Plasmodium* (52), although this prediction was based on the homology restricted to the bromodomain (2% coverage, 48% similarity, *E*-value: 6e–5). Here, we show that BDP5/TAF1 interacts with two other putative TBP-associated factor homologues (TAF 2 and TAF10; (52)) as well as with a WD-repeat protein (PF3D7_1138800), which we found to be homologous to hTAF5 (17% coverage, 47% similarity, *E*-value 2e–20). Combined, these observations suggest that these proteins, together with five other apicomplexan-specific proteins without predicted functions (PF3D7_0824800, PF3D7_1032700, PF3D7_1251800, PF3D7_0514900, PF3D7_1361200) form the elusive TAF complex. Interestingly, we did not find any evidence for interaction of this TAF complex with either of the TBP homologues encoded in the *Plasmodium* genome (PF3D7_0506200, PF3D7_1428800). This observation suggests that this complex does not represent the canonical TFIID complex, but rather an alternative transcription initiation module composed of TAF proteins.

Interestingly, next to these known and putative TAF proteins, two splicing related proteins (SF3B3 and SF3B5) were consistently enriched in our TAF1/BDP5 pulldowns (Figure 4) as well as in earlier H2B.Z_K3/8/13/14/18ac HPPs, where TAF1 was also identified (Figure 1). Since these proteins are members of a major splicing complex (U2 snRNP; (53) but also have been found in association with TAF proteins in the context of the SAGA complex in *Drosophila* and human (47), it is likely that they are genuine component of the TAF1/BDP5 module in *Plasmodium* and they confer extra functionality to this complex.

**Figure 5.**
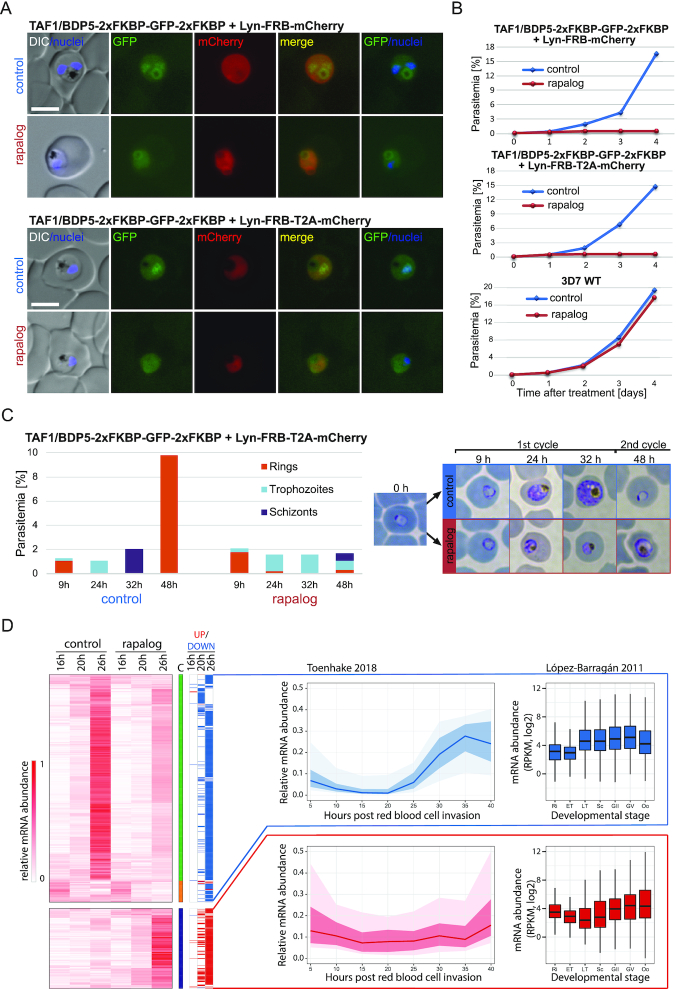
TAF1/BDP5 is a nuclear protein essential for transcription during asexual development. (**A**) Fluorescence and differential interference contrast (DIC) images of parasites in which the endogenous *taf1/bdp5* gene is tagged with a sequence encoding 2xFKBP-GFP-2xFKBP tag (Supplementary Figure S5A) and carrying a plasmid with the Lyn-FRB-mCherry or Lyn-FRB-T2A-mCherry mislocalizer (30). Images were taken 24 h after induction of knock sideways of TAF1/BDP5 with rapalog and compared to controls grown without rapalog. Size bars: 5 um; DIC: differential interference contrast; nuclei: DAPI; merge: merged green (GFP) and red (mCherry) channels. (**B**) Flow cytometry-based growth curve of the above described parasites as well as WT 3D7 parasites over a 5-day period in presence (red line) or absence of rapalog (blue line). For two additional replicates see Supplementary Figure S5C. (**C**) Giemsa staining-based staging of synchronized TAF1/BDP5 knock sideways parasites (TAF1/BDP5-2xFKBP-GFP-2xFKBP with Lyn-FRB-T2A-mCherry mislocalizer) grown in presence or absence (control) of rapalog after the time indicated. Bar graph depicts the proportion of red blood cells infected with different intraerythrocytic stages of parasites (parasitemia). Representative images of parasites are shown. These results are representative of three independent experiments. (**D**) Heatmap depicting the relative gene expression levels of genes that display a min of 2-fold change in their mRNA abundance in TAF1/BDP5 deficient parasites at any of the stages of development (UP/DOWN column). Proportion of sums of RPKM values were clustered by *K*-means clustering (column ‘C’) and within the clusters further ordered by hierarchical clustering. The line graphs depict the median (line), 25–75th percentile (dark shading) and 10th–90th percentile (light shading) of the relative gene expression over the red blood cell cycle as proportion of sums of RPKM values. The boxplots depict the mRNA abundance as quantile normalized log2(RPKM) values in the red blood cell cycle as well as in gametocytes and ookinetes. Ri, rings; ET, early trophozoites; LT, late trophozoites; Sc, schizonts; GII, stage II gametocytes; GV, stage V gametocytes; Oo, ookinetes.

In order to gain insight into the function of TAF1/BDP5 we attempted to disrupt its coding sequence, using SLI-TGD, a method permitting for the selection of parasites with a disrupted locus (30). While transfection of the episomal construct was successful, we consistently failed to obtain parasites after G418 selection, in which the construct would be integrated to the genome (data not shown), hinting towards an essential function of the gene. Hence, instead we generated transgenic parasite lines in which the endogenous TAF1/BDP5 gene was tagged with 2xFKBP-GFP-2xFKBP (Supplementary Figure S5A) and carrying an episomal construct expressing either Lyn-FRB-mCherry or Lyn-FRB-T2A-mCherry mislocalizer. These mislocalizers conditionally tether the FKBP-tagged TAF1/BDP5 to the parasite plasma membrane upon rapalog addition (30). Fluorescent life microscopy imaging of the tagged parasites as well as TAF1/BDP5-AID-GFP parasites we used for the IPs (Supplementary Figure S4) revealed weak expression and preferential nuclear localization of the tagged TAF1/BDP5 in particular at ring and trophozoite stages (Figure 5A, Supplementary Figure S5B,E). As expected, upon rapalog addition the nuclear localization of the 2xFKBP-GFP-2xFKBP tagged BDP5/TAF1 protein substantially reduced and no BDP5 above the cellular background was detectable in the nucleus (n = 10 cells compared to n = 10 controls) as assessed by measuring the fluorescence intensity over a cross-section of the cell (Figure 5A, Supplementary Figure S5E). As a consequence of TAF1/BDP5 mislocalization, rapalog-treated parasites displayed a severe growth defect while untreated transgenic parasites as well as rapalog treated WT parasites displayed normal growth characteristics (Figure 5B, Supplementary Figure S5C), suggesting that TAF1/BDP5 is essential for asexual replication. To further characterize this phenotype, we treated synchronized ring stage TAF1/BDP5-FKBP-GFP + Lyn-FRB-T2A-mCherry parasites with rapalog and followed their development using Giemsa staining and light microscopy. While untreated parasites developed through trophozoite and schizonts stages and after 48 invaded fresh red blood cells, most treated parasites stalled in a trophozoite-like form and only few parasites progressed to schizont and even fewer reached the ring stage (Figure 5C). In order to gain insight to why these BDP5/TAF1 deficient parasites stall in development, we performed RNA-seq analysis of rapalog treated and untreated TAF1/BDP5-FKBP-GFP + Lyn-FRB-T2A-mCherry parasites 2, 6, 12 h after inactivation of BDP5/TAF1 by knock-sideways (corresponding to 16, 20, 26 h post infection). To test for a potential effect of rapalog treatment, we also carried out the 12 h rapalog treatment with the TAF1/BDP5-FKBP-GFP parasites that do not express the mislocalizer. As expected, these control parasites did not display transcriptional changes upon rapalog treatment (Supplementary Figure S5D). In contrast, TAF1/BDP5-FKBP-GFP + Lyn-FRB-T2A-mCherry parasites displayed increasing degree of misregulation in their gene expression 6 and 12 h after inactivation of TAF1/BDP5 (Supplementary Figure S5D, Table S6). Most differentially expressed genes showed decreased RNA abundance in the TAF1/BDP5 deficient parasites (560 genes with a minimum of 2-fold decrease in any of the stages, Figure 5D). Most of these down-regulated genes normally start expressing at 20–25 h post infection coinciding with the time knock sideways took effect (Figure 5D). This observation is consistent with the predicted function of TAF1/BDP5 complex in gene expression initiation and explains the observed lack of developmental progression of the rapalog treated parasites. Interestingly, we also detected a smaller group of genes that displayed increased mRNA abundance in TAF1/BDP5 deficient parasites (213 genes, Figure 5D). Most of these genes are express at low levels in blood stage parasites and some are primarily expressed in late gametocytes, raising the intriguing possibility that either the depletion of TAF1/BDP5 has led to the loss of expression of a repressor keeping these genes at bay or it might be directly involved in the repression of these genes in asexual blood stages. This possibility however needs to be further validated.

Altogether, these experiments identify members of the elusive TAF module and demonstrate the essentiality of a major component of this module, TAF1/BDP5, for gene expression regulation and the progression of parasites through the disease-causing, asexual blood-stage replication.

## DISCUSSION

In this manuscript, we present the first large-scale characterisation of the interaction between histone tail modifications and associated reader proteins in *P. falciparum* using histone peptide pull-down coupled to quantitative mass-spectrometry (Figure 1). Our analysis revealed that many of these reader proteins reside in multiprotein complexes, also containing ‘writers’ of histone modifications or chromatin remodelling enzymes (Figure 4). Thereby, they could mediate cross-talk between modifications or provide a biological ‘interpretation’ of histone PTMs by alterations in the chromatin structure upon recruitment. A prime and well-conserved example of such crosstalk is the placement of the H3K9ac modification by the GCN5/ADA2 complex, which is recruited to the H3K4me2/3 modification via a tudor-domain protein (44). Interestingly, we find that in *Plasmodium* GCN5/ADA2 is recruited to H3K4me2/3 by a parasite-specific PhD-finger-containing protein (PHD1, Figure 3) that could mediate a similar cross-talk.

Notably, many of these complexes contain more than one reader protein (e.g. BDP1/BDP2, GCN5/PHD1 or TAF1/BDP3, Figure 4). The reason for the presence of multiple readers in a single complex raises some interesting hypotheses. On the one hand, the combination of multiple readers could increase specificity of recruitment to specific genomic locations containing two different histone modifications via multiple (weaker) interactions. The weak interaction of TAF1/BDP5 with the penta-acetylated H2B.Z observed in our HPP assay (Figure 1) could indicate that the interaction with the BDP3 or the BDP1/BDP2 complexes might be required to reach sufficient affinity to recognise a specific combination of modifications. Alternatively, the combination of two different reader proteins could confer broad specificity towards multiple histone marks and explain the modification-level-dependent recognition as we observed for BDP1/BDP2 complex (Figure 2). Further increasing the level of complexity is the observed high degree of connectivity between the distinct, but highly interlinked epigenetic protein modules and the multiple distinct ‘flavours’ of each module. Our reciprocal pull-downs for PHD1 and PHD2 suggest that indeed the histone acetyl-transferase GCN5/ADA2 complex exists in at least two distinct flavours (Figure 3D). Interestingly, the PhD-fingers of PHD2 do not contain the critical residues for H3K4me3 binding. Instead this protein contains 12 predicted transmembrane domains suggesting that the PHD2-containing complex might (also) acetylate proteins in a non-chromatin context or associate with the nuclear membrane. Alternatively, this complex could be recruited to the chromatin via a different recruiter protein (histone or DNA binding) present within the complex, or indirectly via interaction with other complexes (e.g. BDP4, BDP1/2; Figure 4).

Last but not least, many of these complexes contain transcription factors (e.g. Ap2-I, SIP2, PF3D7_1449500). These could either by themselves be sufficient to recruit epigenetic complexes to specific DNA elements, as has been demonstrated for AP2-I (25). Alternatively, they could increase the affinity of recruitment to specific chromatin regions that contain a combination of both a DNA element and a certain PTM. In summary, while recruitment of some epigenetic complexes might be achieved by a prominent reader PTM interaction (e.g. H3K9me3-HP1, H3K4me3-PHD1), most of these complexes are likely recruited via the combined action of various binding events (e.g. two reader proteins, reader and transcription factor, etc.) and these later complexes might even contain slightly different sets of proteins depending on the combination of (epi)genomic features that are present at the given locus.

Reader protein-PTM interactions can be disrupted by small molecule inhibitors (54) and hence serve as promising targets for antimalarial drug development. While many of the histone modifications (e.g. H3K9ac, H4ac,) are conserved between the parasites and its human host, other modifications appear to be specific to the parasite (e.g. H4K31, (55)). Perhaps readers of these unique modifications could be more selectively targeted. Of particular interest are the heavily acetylated histone tails of the parasite-specific histone variants H2A.Z and H2B.Z, which we found to be recognized by several bromodomain and (potentially) an EELM2-domain-containing protein (Figure 1). Notably, two third of the (putative) reader proteins were found to be essential/necessary for asexual replication in at least one of the two large scale mutagenesis screens (56,57); Supplementary Table S1), suggesting that inhibiting their interaction with PTMs could have a lethal effect as well. For example, BDP1 is essential for regulation of invasion related genes (57) or depletion of HP1 prevents mitotic proliferation of blood stage parasites and disrupts mutually exclusive expression and antigenic variation (18). Further, we here show that in absence of functional TAF1/BDP5, which is an integral part of the elusive TAF complex, asexual parasites halt in development (Figure 5) (58). Importantly, bromodomain inhibitors predicted or shown to bind eukaryotic bromodomains and inhibit their activity, exhibit at best moderate growth inhibition against asexual parasites (i.e. in the micromolar range; (59)). The fact that these inhibitors that were mainly developed against human reader proteins are not efficient against *Plasmodium*, can be interpreted as an encouraging sign that *Plasmodium*-selective reader inhibitors might indeed exist. Detailed structural studies could enable identification of inhibitors against the parasite proteins and increase their potency and selectivity (e.g. (60)). Interestingly, in our study we furthermore identified putative novel reader proteins (e.g. PF3D7_1140700, _0519600, _0707200) which could be exploited as drug target. Finally, it is important to note that the effectiveness and specificity of inhibitors to a drug target can be modulated by the molecular interactions within a protein complex (61). To this end, it is encouraging that most of the complexes identified in this study show little resemblance to human complexes and at least 50% of the proteins residing in these complexes are parasite-specific proteins (Figure 4). Hence the unique composition of *Plasmodium* complexes might further increase the selectivity of potential antimalarial drugs targeting reader proteins. To exploit this possibility, chemo-proteomics approaches (62) based on the histone peptide pulldown described in this study could be used to measure the affinity of compounds against intact reader proteins in a much more natural context (carrying posttranslational modifications and in the context of protein complexes). Hence, our data not only provide insights into epigenetic complexes of an ancient eukaryotic organism, but delivers tools and information for the development of epi-drugs against a deadly human pathogen.

## DATA AVAILABILITY

All proteomics data has been deposited to the ProteomeXchange Consortium (http://www.proteomexchange.org/) using the PRIDE partner repository (63). These data are available with data set identifier PXD010644.

RNA-seq data of the TAF1/BDP5 KS parasites has been deposited to GEO database https://www.ncbi.nlm.nih.gov/geo/ and is available under GSE138499 identifier.

## Supplementary Material

gkz1044_Supplemental_Files
