# Supplementary material for: Epigenetic reader complexes of the human malaria parasite, Plasmodium falciparum
Source: Nucleic Acids Res. 2019 Nov 15;47(22):11574–88. doi: 10.1093/nar/gkz1044 (PMC7145593; doi:10.1093/nar/gkz1044)
Supplement: gkz1044_Supplemental_Files [file gkz1044_supplemental_files.zip › Supplementary_Information.pdf]

# SUPPLEMENTARY INFORMATION

## Epigenetic reader complexes of the human malaria parasite *Plasmodium falciparum*

Wieteke Anna Maria Hoeijmakers,[1,*] Jun Miao,[2,5] Sabine Schmidt,[3] Christa Geeke Toenhake,[1] Sony Shrestha,[2] Jeron Venhuizen,[1] Rob Henderson,[1,6] Jakob Birnbaum,[3] Sonja Ghidelli-Disse,[4] Gerard Drewes,[4] Liwang Cui,[2,5] Hendrik Gerard Stunnenberg,[1] Tobias Spielmann,[3] and Richárd Bártfai [1,*]

**Supplementary Figure S1:** Histone Peptide Pulldown enables identification of histone readers and associated proteins

**Supplementary Figure S2:** Bromodomain readers exhibit moderate site-specificity and are recruited in an acetylation level-dependent manner

**Supplementary Figure S3:** The HAT module of a SAGA-like complex is recruited to H3K4me2/me3 via an unconventional PhD-finger containing reader

**Supplementary Figure S4:** Validation of transgenic parasite lines used for co-immunoprecipitation experiments

**Supplementary Figure S5:** TAF1/BDP5 is a nuclear protein essential for asexual development

**Supplementary Material and Methods**

# Figure S1

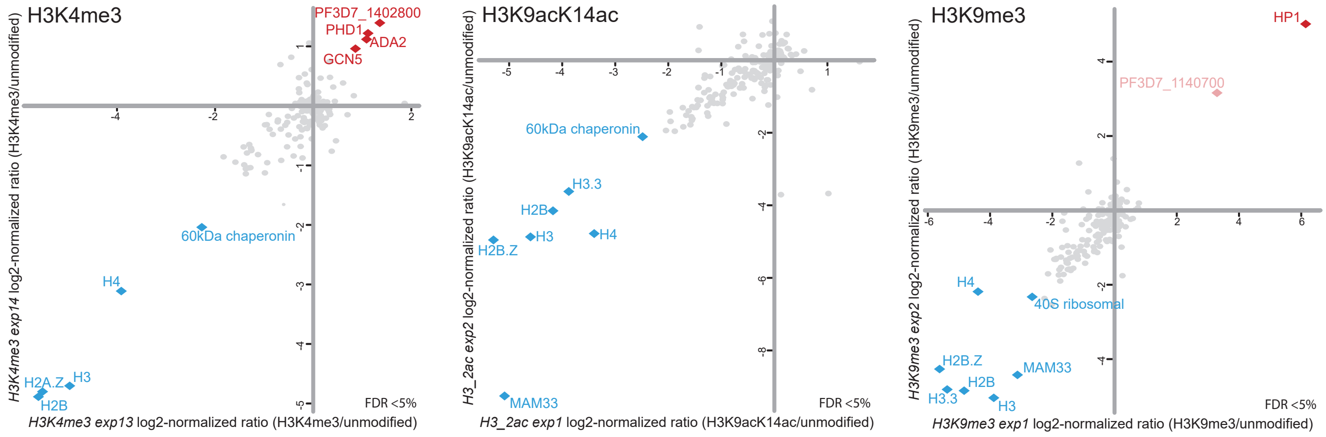

H3K4me3
PF3D7_1402800
PHD1
ADA2
GCN5
60kDa chaperonin
H4
H2A.Z
H3
H2B
FDR <5%
H3K4me3 exp13 log2-normalized ratio (H3K4me3/unmodified)
H3K4me3 exp14 log2-normalized ratio (H3K4me3/unmodified)
H3K9acK14ac
60kDa chaperonin
H3.3
H2B
H3
H4
H2B.Z
MAM33
FDR <5%
H3_2ac exp1 log2-normalized ratio (H3K9acK14ac/unmodified)
H3_2ac exp2 log2-normalized ratio (H3K9acK14ac/unmodified)
H3K9me3
HP1
PF3D7_1140700
H4
40S ribosomal
H2B.Z
MAM33
H2B
H3.3
H3
FDR <5%
H3K9me3 exp1 log2-normalized ratio (H3K9me3/unmodified)
H3K9me3 exp2 log2-normalized ratio (H3K9me3/unmodified)

**Supplementary Figure S1: Histone Peptide Pulldown enables identification of histone readers and associated proteins**

Scatterplots of log2 normalized ratios of protein enrichments (modified/unmodified) in histone peptide pull downs using H3K9me3, K3K9/14ac and H3K4me3 as well as unmodified control peptides. Axes are auto-scaled to display all enriched or repelled proteins. Proteins significantly enriched/repelled (FDR <5%) in both experiments are highlighted with red or blue diamonds, respectively. See also **Figure 1** and **Table S2**.

# Figure S2

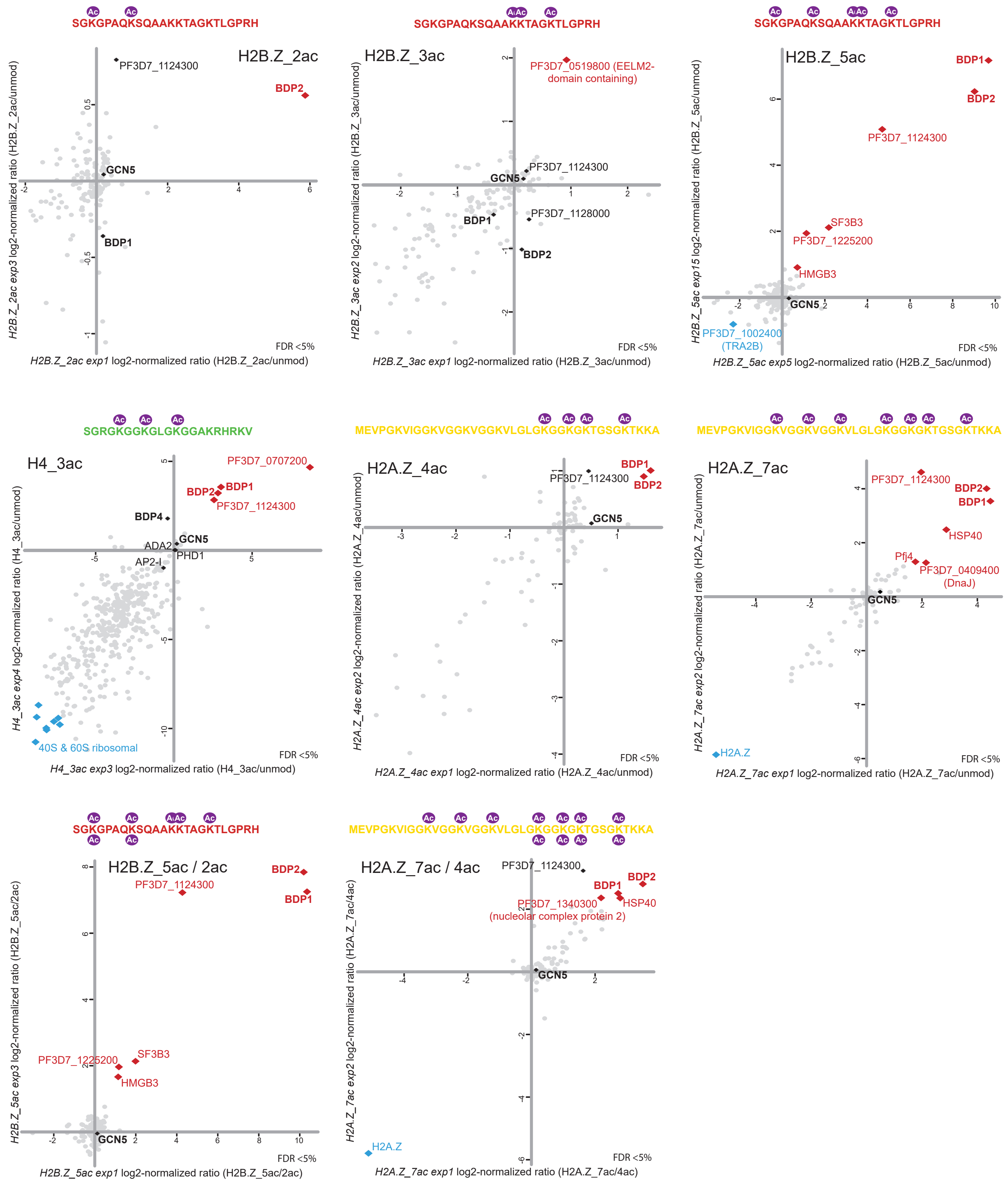
SGKGPAQKSQAAKKTAGKTLGPRH
H2B.Z_2ac
PF3D7_1124300
BDP2
GCN5
BDP1
FDR <5%
H2B.Z_2ac exp1 log2-normalized ratio (H2B.Z_2ac/unmod)
H2B.Z_2ac exp3 log2-normalized ratio (H2B.Z_2ac/unmod)
SGKGPAQKSQAAKKTAGKTLGPRH
H2B.Z_3ac
PF3D7_0519800 (EELM2-domain containing)
PF3D7_1124300
GCN5
BDP1
PF3D7_1128000
BDP2
FDR <5%
H2B.Z_3ac exp1 log2-normalized ratio (H2B.Z_3ac/unmod)
H2B.Z_3ac exp2 log2-normalized ratio (H2B.Z_3ac/unmod)
SGKGPAQKSQAAKKTAGKTLGPRH
H2B.Z_5ac
BDP1
BDP2
PF3D7_1124300
SF3B3
PF3D7_1225200
HMGB3
GCN5
PF3D7_1002400 (TRA2B)
FDR <5%
H2B.Z_5ac exp5 log2-normalized ratio (H2B.Z_5ac/unmod)
H2B.Z_5ac exp15 log2-normalized ratio (H2B.Z_5ac/unmod)
SGRGKGGKGLGKGGAKRHRKV
H4_3ac
PF3D7_0707200
BDP2
BDP1
PF3D7_1124300
BDP4
GCN5
ADA2
PHD1
AP2-I
40S & 60S ribosomal
FDR <5%
H4_3ac exp3 log2-normalized ratio (H4_3ac/unmod)
H4_3ac exp4 log2-normalized ratio (H4_3ac/unmod)
MEVPGKVIGGKVGGKVGGKVLGLGKGGKGKTGSGKTKKA
H2A.Z_4ac
BDP1
PF3D7_1124300
BDP2
GCN5
FDR <5%
H2A.Z_4ac exp1 log2-normalized ratio (H2A.Z_4ac/unmod)
H2A.Z_4ac exp2 log2-normalized ratio (H2A.Z_4ac/unmod)
MEVPGKVIGGKVGGKVGGKVLGLGKGGKGKTGSGKTKKA
H2A.Z_7ac
PF3D7_1124300
BDP2
BDP1
HSP40
Pfj4
PF3D7_0409400 (DnaJ)
GCN5
H2A.Z
FDR <5%
H2A.Z_7ac exp1 log2-normalized ratio (H2A.Z_7ac/unmod)
H2A.Z_7ac exp2 log2-normalized ratio (H2A.Z_7ac/unmod)
SGKGPAQKSQAAKKTAGKTLGPRH
H2B.Z_5ac / 2ac
BDP2
PF3D7_1124300
BDP1
SF3B3
PF3D7_1225200
HMGB3
GCN5
FDR <5%
H2B.Z_5ac exp1 log2-normalized ratio (H2B.Z_5ac/2ac)
H2B.Z_5ac exp3 log2-normalized ratio (H2B.Z_5ac/2ac)
MEVPGKVIGGKVGGKVGGKVLGLGKGGKGKTGSGKTKKA
H2A.Z_7ac / 4ac
PF3D7_1124300
BDP2
BDP1
PF3D7_1340300 (nucleolar complex protein 2)
HSP40
GCN5
H2A.Z
FDR <5%
H2A.Z_7ac exp1 log2-normalized ratio (H2A.Z_7ac/4ac)
H2A.Z_7ac exp2 log2-normalized ratio (H2A.Z_7ac/4ac)

**Supplementary Figure S2: Bromodomain readers exhibit moderate site-specificity and are recruited in an acetylation level-dependent manner**

Scatterplots of log2 normalized ratios of protein enrichments in histone peptide pull downs for H2B.Z_2ac (H2B.Z_K3/8ac), H2B.Z_3ac (H2B.Z_13/14/18ac), H2B.Z_5ac (H2B.Z_K3/8/13/14/18ac), H4_3ac (H4K5/8/12ac), H2A.Z_4ac (H2A.Z_K25/28/30/35ac) and H2A.Z_7ac (H2A.Z_K11/15/19/25/28/30/35ac) over unmodified control peptides as well as H2B.Z_5ac over 2ac and H2A.Z_7ac over 4ac peptide. Axes are auto-scaled to display all enriched or repelled proteins. Histone peptide sequences and modifications are depicted above the scatterplot. Proteins significantly enriched/repelled (FDR <5%) in both experiments are highlighted with red or blue diamonds, respectively. See also **Figure 2** and **Table S2**.

# Figure S3

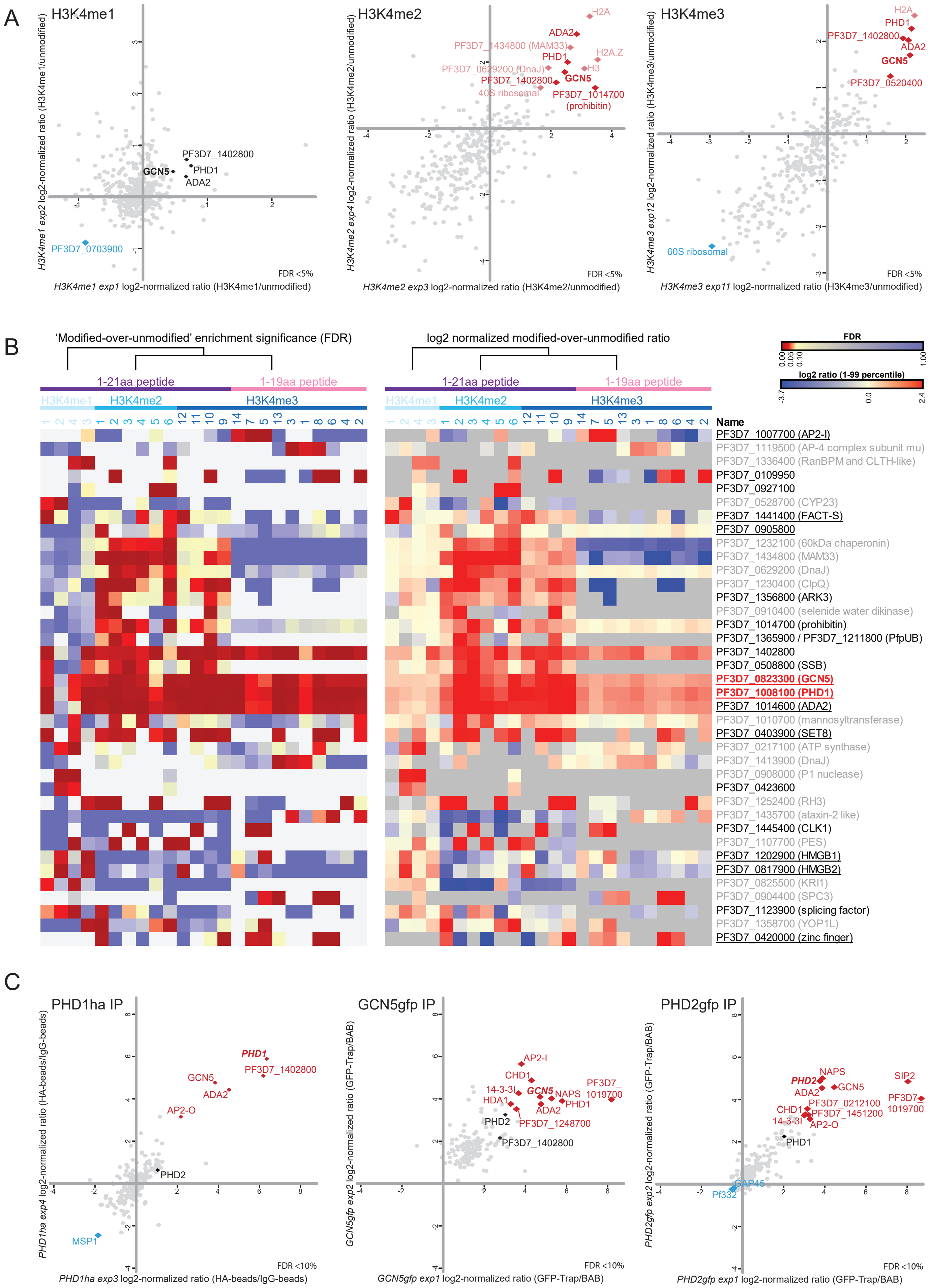
A
H3K4me1
H3K4me2
H3K4me3
GCN5
PF3D7_1402800
PHD1
ADA2
PF3D7_0703900
H2A
H2A.Z
H3
PF3D7_1434800 (MAM33)
PF3D7_0629200 (DnaJ)
40S ribosomal
PF3D7_1014700 (prohibitin)
PF3D7_0520400
60S ribosomal
FDR <5%
H3K4me1 exp1 log2-normalized ratio (H3K4me1/unmodified)
H3K4me1 exp2 log2-normalized ratio (H3K4me1/unmodified)
H3K4me2 exp3 log2-normalized ratio (H3K4me2/unmodified)
H3K4me2 exp4 log2-normalized ratio (H3K4me2/unmodified)
H3K4me3 exp11 log2-normalized ratio (H3K4me3/unmodified)
H3K4me3 exp12 log2-normalized ratio (H3K4me3/unmodified)
B
'Modified-over-unmodified' enrichment significance (FDR)
log2 normalized modified-over-unmodified ratio
1-21aa peptide
1-19aa peptide
FDR
log2 ratio (1-99 percentile)
Name
PF3D7_1007700 (AP2-I)
PF3D7_1119500 (AP-4 complex subunit mu)
PF3D7_1336400 (RanBPM and CLTH-like)
PF3D7_0109950
PF3D7_0927100
PF3D7_0528700 (CYP23)
PF3D7_1441400 (FACT-S)
PF3D7_0905800
PF3D7_1232100 (60kDa chaperonin)
PF3D7_1434800 (MAM33)
PF3D7_0629200 (DnaJ)
PF3D7_1230400 (ClpQ)
PF3D7_1356800 (ARK3)
PF3D7_0910400 (selenide water dikinase)
PF3D7_1014700 (prohibitin)
PF3D7_1365900 / PF3D7_1211800 (PfpUB)
PF3D7_1402800
PF3D7_0508800 (SSB)
PF3D7_0823300 (GCN5)
PF3D7_1008100 (PHD1)
PF3D7_1014600 (ADA2)
PF3D7_1010700 (mannosyltransferase)
PF3D7_0403900 (SET8)
PF3D7_0217100 (ATP synthase)
PF3D7_1413900 (DnaJ)
PF3D7_0908000 (P1 nuclease)
PF3D7_0423600
PF3D7_1252400 (RH3)
PF3D7_1435700 (ataxin-2 like)
PF3D7_1445400 (CLK1)
PF3D7_1107700 (PES)
PF3D7_1202900 (HMGB1)
PF3D7_0817900 (HMGB2)
PF3D7_0825500 (KRI1)
PF3D7_0904400 (SPC3)
PF3D7_1123900 (splicing factor)
PF3D7_1358700 (YOP1L)
PF3D7_0420000 (zinc finger)
C
PHD1ha IP
GCN5gfp IP
PHD2gfp IP
PHD1
GCN5
ADA2
AP2-O
PHD2
MSP1
AP2-I
CHD1
14-3-3I
HDA1
NAPS
PF3D7_1019700
PF3D7_1248700
PF3D7_1402800
SIP2
PF3D7_0212100
PF3D7_1451200
GAP45
Pf332
FDR <10%
PHD1ha exp3 log2-normalized ratio (HA-beads/IgG-beads)
PHD1ha exp4 log2-normalized ratio (HA-beads/IgG-beads)
GCN5gfp exp1 log2-normalized ratio (GFP-Trap/BAB)
GCN5gfp exp2 log2-normalized ratio (GFP-Trap/BAB)
PHD2gfp exp1 log2-normalized ratio (GFP-Trap/BAB)
PHD2gfp exp2 log2-normalized ratio (GFP-Trap/BAB)

**Supplementary Figure S3: The HAT module of a SAGA-like complex is recruited to H3K4me2/me3 via an unconventional PhD-finger containing reader**

**A)** Scatterplots of log2 normalized ratios of protein enrichments in histone peptide pull downs: H3K4me1, me2 and me3 over unmodified peptide corresponding to the 21 amino acid N-terminal sequence of *P. falciparum* H3.3. Axes are auto-scaled to display all enriched or repelled proteins. The components of the GCN5/ADA2 core complex are highlighted (note that their enrichment on the H3K4me1 pull down is not significant).

**D)** Heat map displaying the false discovery rate (FDR) or log2 normalized ratios for proteins identified in histone peptide pull downs: H3K4me1, me2 and me3 over unmodified peptide corresponding to the first 19 or 21 amino acid of *P. falciparum* H3.3 as indicated. Proteins significantly enriched with FDR <5% in at least 20% of the reactions per PTM, with a minimum of 2 reactions are listed. Columns were ordered using a 2-layered hierarchical clustering approach. Rows were ranked based on hierarchical clustering on FDR values. Proteins that the authors considered likely contaminants based on proven non-nuclear localization or predicted function are listed in grey. See also **Figure 3** and **Table S2**.

**C)** Scatterplots of log2 normalized ratio's are plotted for technical replicate experiments of GFP-Trap-over-BAB control (GCN5 and PHD2) or HAbead-over-IgGbead control (PHD1). Only proteins identified by at least 2 peptides (of which minimally 1 unique peptide) in both reactions are included in the plot. Significant outliers with FDR <10% are labelled in the plot in blue (depleted) or red (enriched). Proteins significantly enriched or depleted in GFP- and HA-control experiments were removed from the plots. The SAGA-like components PHD1, PHD2 and PF3D7_1402800 are highlighted black when identified, but non-significant. See also **Figure 3** and **Table S2**.

# Figure S4

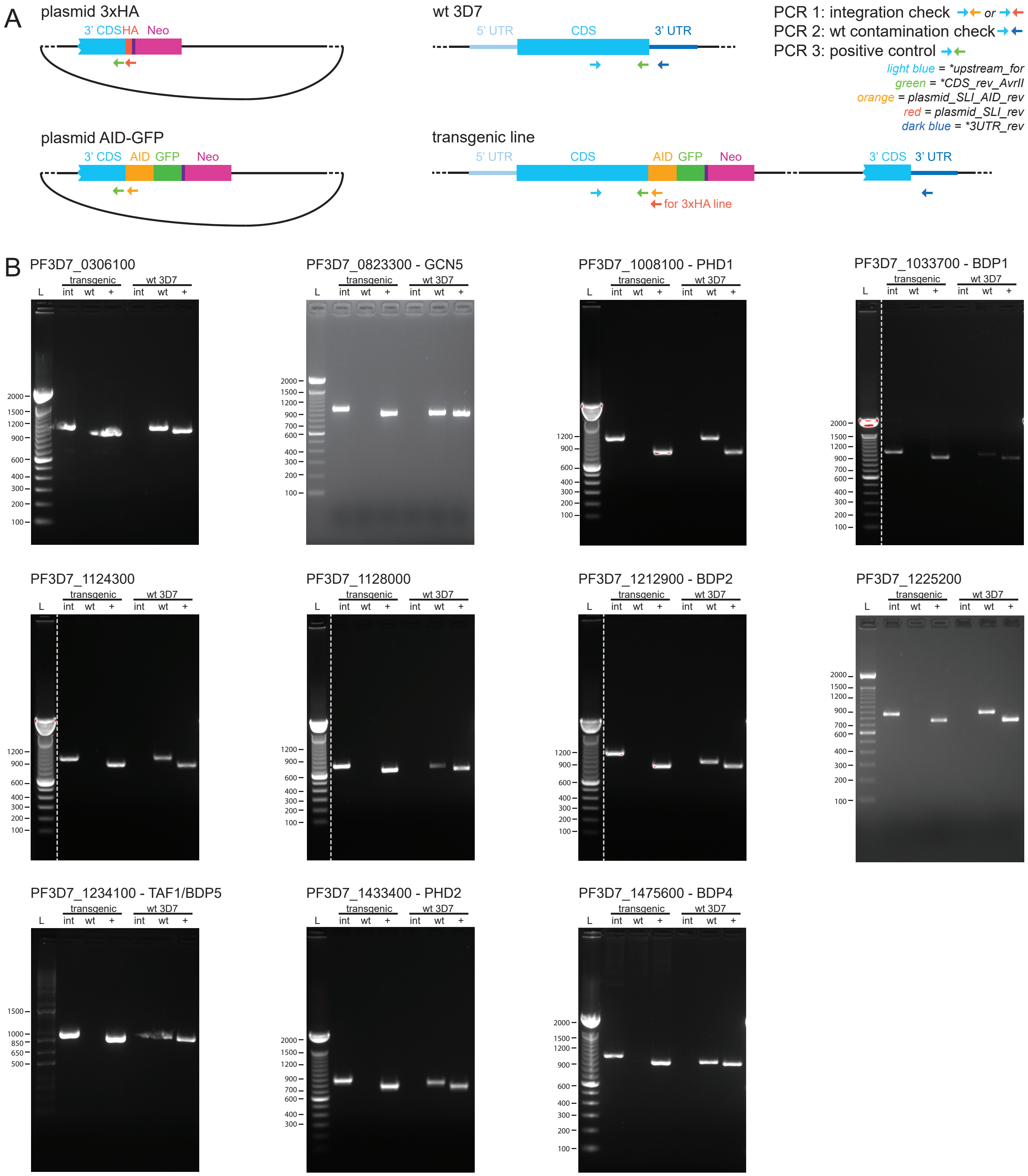

A
plasmid 3xHA
3' CDS HA Neo
plasmid AID-GFP
3' CDS AID GFP Neo
wt 3D7
5' UTR CDS 3' UTR
transgenic line
5' UTR CDS AID GFP Neo 3' CDS 3' UTR
for 3xHA line
PCR 1: integration check or
PCR 2: wt contamination check
PCR 3: positive control
light blue = *upstream_for
green = *CDS_rev_AvrII
orange = plasmid_SLI_AID_rev
red = plasmid_SLI_rev
dark blue = *3UTR_rev
B
PF3D7_0306100
PF3D7_0823300 - GCN5
PF3D7_1008100 - PHD1
PF3D7_1033700 - BDP1
PF3D7_1124300
PF3D7_1128000
PF3D7_1212900 - BDP2
PF3D7_1225200
PF3D7_1234100 - TAF1/BDP5
PF3D7_1433400 - PHD2
PF3D7_1475600 - BDP4
transgenic
wt 3D7
L int wt + int wt +

**Supplementary Figure S4: Validation of transgenic parasite lines used for co-immunoprecipitation experiments**

**A)** Schematic of 3xHA and AID-GFP SLI plasmids, wild type locus (wt 3D7) and integrated transgenic locus with the location of primers indicated. For each transgenic line, 3 PCR reactions were performed on genomic DNA isolated from the parasites that were selected for integration by geneticin G418 treatment. As a control, the same 3 PCR reactions were performed on genomic DNA extracted from wt 3D7 parasites. PCR 1: test integration of the SLI-tagging plasmid in the correct genomic locus, PCR 2: test for the presence of wt locus and PCR 3: positive control amplifying the 3' CDS region from both the wt and the integrated locus. All primers are listed in **Table S5**.

**B)** PCR product of PCR 1 (int), PCR 2 (wt) and PCR 3 (+) was ran on a 1 - 1.5% agarose/TAE ethidium bromide gel for transgenic lines (left) and control wild type 3D7 (right). Dashed lines highlight gels were the ladder was moved from the left side of the PCR products, to the right side. Overexposed images were obtained from all gels to verify the absence of a wt PCR product in the transgenic lines (pictures not shown) and all transgenic lines were free of detectable wt contamination.

# Figure S5

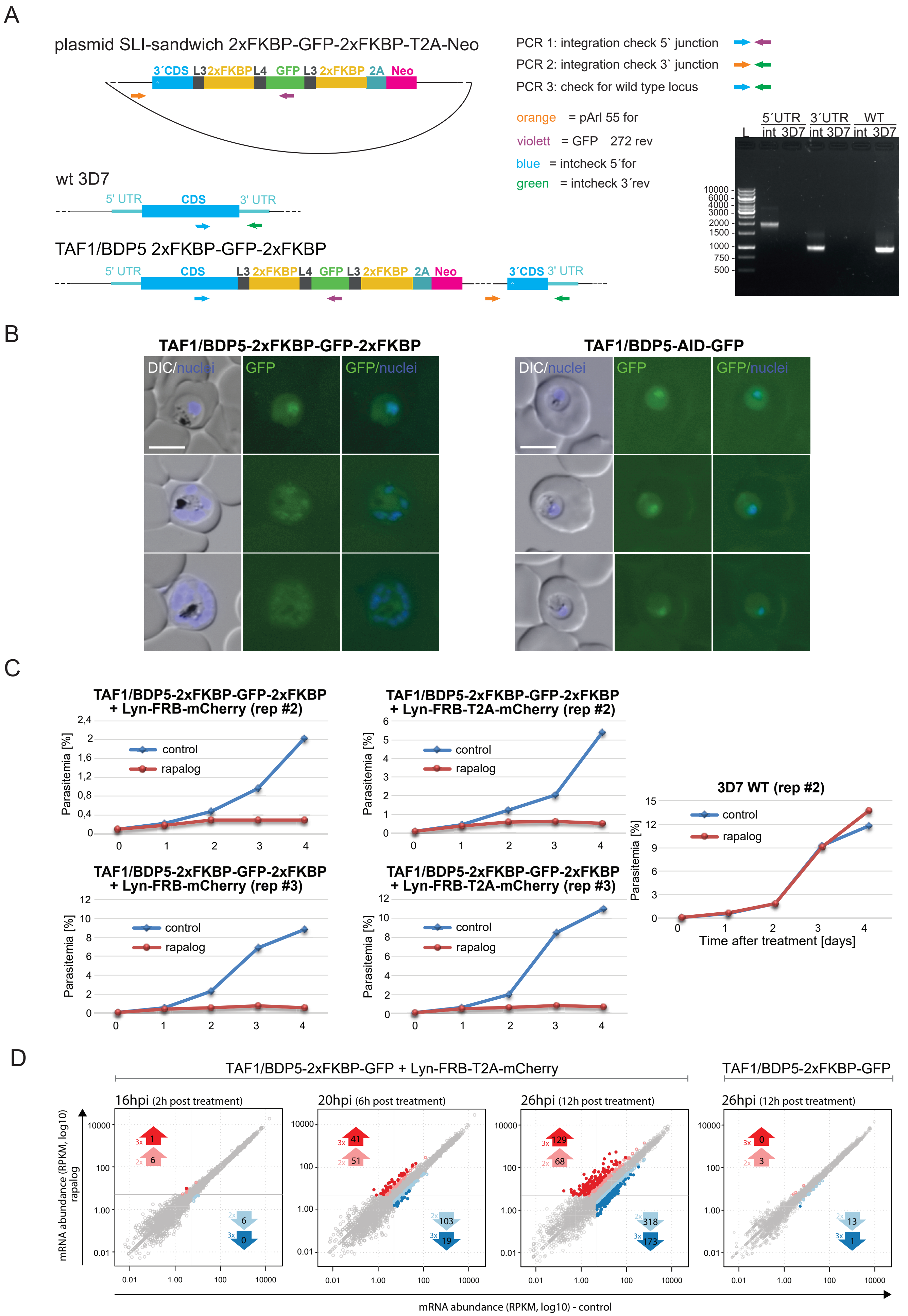
A
plasmid SLI-sandwich 2xFKBP-GFP-2xFKBP-T2A-Neo
3´CDS L3 2xFKBP L4 GFP L3 2xFKBP 2A Neo
PCR 1: integration check 5` junction
PCR 2: integration check 3` junction
PCR 3: check for wild type locus
orange = pArl 55 for
violett = GFP 272 rev
blue = intcheck 5´for
green = intcheck 3´rev
wt 3D7
5' UTR CDS 3' UTR
TAF1/BDP5 2xFKBP-GFP-2xFKBP
5' UTR CDS L3 2xFKBP L4 GFP L3 2xFKBP 2A Neo 3´CDS 3' UTR
5´UTR 3´UTR WT
L int 3D7 int 3D7 int 3D7
10000 6000 4000 3000 2000 1500 1000 750 500
B
TAF1/BDP5-2xFKBP-GFP-2xFKBP
TAF1/BDP5-AID-GFP
DIC/nuclei GFP GFP/nuclei
C
TAF1/BDP5-2xFKBP-GFP-2xFKBP + Lyn-FRB-mCherry (rep #2)
TAF1/BDP5-2xFKBP-GFP-2xFKBP + Lyn-FRB-T2A-mCherry (rep #2)
3D7 WT (rep #2)
TAF1/BDP5-2xFKBP-GFP-2xFKBP + Lyn-FRB-mCherry (rep #3)
TAF1/BDP5-2xFKBP-GFP-2xFKBP + Lyn-FRB-T2A-mCherry (rep #3)
control
rapalog
Parasitemia [%]
Time after treatment [days]
D
TAF1/BDP5-2xFKBP-GFP + Lyn-FRB-T2A-mCherry
TAF1/BDP5-2xFKBP-GFP
16hpi (2h post treatment)
20hpi (6h post treatment)
26hpi (12h post treatment)
26hpi (12h post treatment)
mRNA abundance (RPKM, log10) rapalog
mRNA abundance (RPKM, log10) - control
3x 1 2x 6 2x 6 3x 0
3x 41 2x 51 2x 103 3x 19
3x 129 2x 68 2x 318 3x 173
3x 0 2x 3 2x 13 3x 1

# Figure S5

E

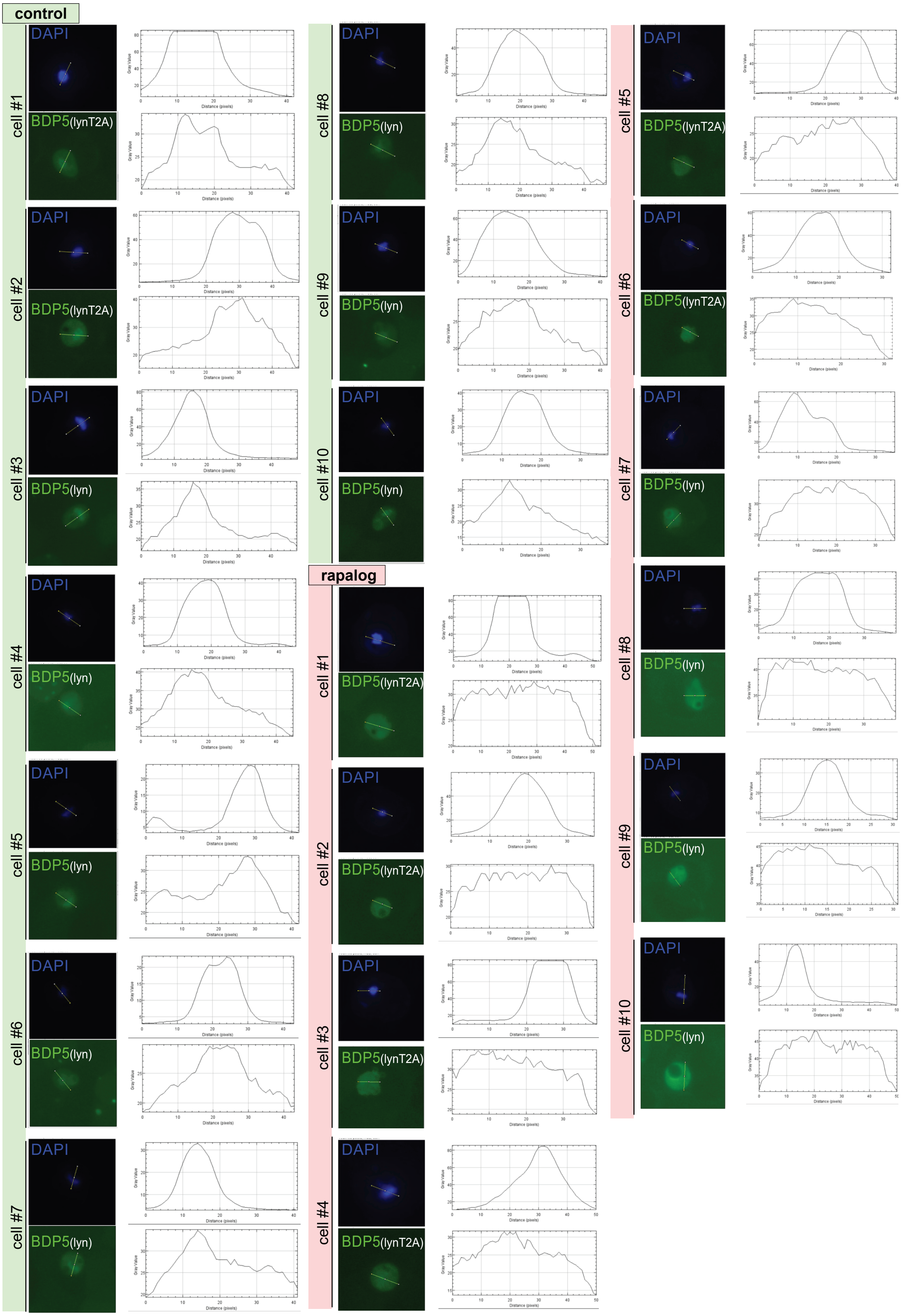
control
rapalog
cell #1
cell #2
cell #3
cell #4
cell #5
cell #6
cell #7
cell #8
cell #9
cell #10
DAPI
BDP5(lynT2A)
BDP5(lyn)
Gray Value
Distance (pixels)

**Supplementary Figure S5: TAF1/BDP5 is a nuclear protein essential for asexual development**

**A)** Schematic of the SLI-sandwich 2xFKBP-GFP-2xFKBP-T2A-Neo plasmid, wild type *taf1/bdp5* locus (wt 3D7) and integrated transgenic *taf1/bdp5* locus with the location of primers indicated. PCR reactions were performed on genomic DNA isolated from the parasites that were selected for integration by geneticin G418 treatment as well as genomic DNA extracted from wt 3D7 parasites. PCR 1: test integration of the plasmid to the *taf1/bdp5* locus at the 5' end; PCR 2: test integration of the plasmid to the *taf1/bdp5* locus at the 3' end and PCR 3: test for the presence of wt *taf1/bdp5* locus. PCR products were separated on a 1 - 1.5% agarose/TAE ethidium bromide gel.

**B)** Fluorescence and differential interference contrast (DIC) images of parasites in which the endogenous *taf1/bdp5* gene is tagged with a sequence encoding 2xFKBP-GFP-2xFKBP tag (**Figure S5A**) or AID-GFP tag (**Figure S4**). Size bars: 5 um; DIC: differential interference contrast; nuclei: DAPI.

**C)** Flow cytometry-based growth curve of parasites in which the endogenous *taf1/bdp5* gene is tagged with a sequence encoding 2xFKBP-GFP-2xFKBP tag (**Figure S5A**) and carrying a plasmid with the Lyn-FRB-mCherry or Lyn-FRB-T2A-mCherry mislocalizer (Birnbaum et al., 2017) over a 5-day period in presence (red line) or absence of rapalog (blue line). Graphs depicts two additional replicates next to the one displayed on **Figure 5** as well as one additional replicates for wild type 3D7 parasites growing in presence or absence of rapalog.

**D**) Scatter plots depicting the mRNA abundance as measured by RNA-seq (RPKM, log10) in TAF1/BDP5-2xFKBP-GFP + Lyn-FRB-T2A-mCherry as well as TAF1/BDP5-2xFKBP-GFP rapalog treated and untreated, control parasites 2, 6 or 12h after induction of knock sideway. Genes with 2-3 or higher than 3-fold changes in their abundance are coloured (UP – red, DOWN – blue). The number of genes with differential expression (2-3x or >3x) is indicated in the red and blue arrows.

**E)** Semiquantitative assessment of the efficiency of knock sideways by measuring the fluorescence intensity (graphs) from microscopy images of the TAF1/BDP5-2xFKBP-GFP + Lyn-FRB-T2A-mCherry (**Figure S5A & B**) along a cross section through the cell including the nucleus (evident from the DAPI channel). In the untreated parasites the GFP signal matches the intensity profile of the DAPI channel, indicating concentration of BDP5 in the nucleus. On the contrary, after addition of rapalog (knock sideways) GFP peak correlating with the DAPI signal was undetectable. N = 10 cells for control and rapalog treated parasites, respectively.

# Supplementary Material and Methods

*Plasmodium falciparum culturing*

Blood-stage *P. falciparum* parasites were maintained in a shaking, semi-automated 37 °C incubator under low oxygen conditions (gas composition 3% $O_2$, 4% $CO_2$ and 93% $N_2$) in human O+ red blood cells at 5% hematocrit in standard RPMI medium supplemented with 10% human serum or 0.5% Albumax (Life Technologies) and 0.2% NaHCO3. Wild-type 3D7 parasites were grown in the absence of antibiotics, while integrated transgenic lines (see below) were maintained in the presence of 400 µg/ml Geneticin G-418 Sulpate (Geneticin Selective Antibiotic (G418 Sulpate), Gibco, Thermo Fisher Scientific, cat# 11811031, dissolved to 50 mg/ml in MQ). Upon culture expansion for nuclear collections, parasites were transferred to 175 $cm^2$ tissue culture flasks and cultured in a mixed-gas standing incubator (gas composition 3% $O_2$, 4% $CO_2$ and 93% $N_2$). The parasites were grown for 3 to 4 days in 50 ml culture volumes with low hematocrit (1.25% and lower) to high mixed-stage asexual parasitemia. Media was changed on demand, either once or twice per 24 h, depending on hematocrit concentration and parasitemia levels.

*Parasite Strains*

| **Name:** | **Description:** | **Normal medium with:** |
|---|---|---|
| *P. falciparum* 3D7 | wild-type *P. falciparum* strain obtained from the Swiss Tropical and Public Health Institute (Basel, Switzerland - a kind gift from Dr. T. Voss) (1) | - |
| *P. falciparum* 3D7 PF3D7_0306100-AID-GFP | *P. falciparum* 3D7 after transfection and confirmed genomic integration of plasmid SLI_NEO_PF3D7_0306100_AID_GFP_GlmS | medium supplemented with 400 µg/ml Geneticin G-418 Sulpate |

| | | |
|---|---|---|
| *P. falciparum* 3D7 PF3D7_0823300-AID-GFP GCN5 | *P. falciparum* 3D7 after transfection and confirmed genomic integration of plasmid SLI_NEO_PF3D7_0823300_AID_GFP_GlmS GCN5 | medium supplemented with 400 µg/ml Geneticin G-418 Sulpate |
| *P. falciparum* 3D7 PF3D7_1008100-3xHA PHD1 | *P. falciparum* 3D7 after transfection and confirmed genomic integration of plasmid SLI_NEO_PF3D7_1008100_3xHA_GlmS PHD1 | medium supplemented with 400 µg/ml Geneticin G-418 Sulpate |
| *P. falciparum* 3D7 PF3D7_1033700-AID-GFP BDP1 | *P. falciparum* 3D7 after transfection and confirmed genomic integration of plasmid SLI_NEO_PF3D7_1033700_AID_GFP_GlmS BDP1 | medium supplemented with 400 µg/ml Geneticin G-418 Sulpate |
| *P. falciparum* 3D7 PF3D7_1124300-AID-GFP | *P. falciparum* 3D7 after transfection and confirmed genomic integration of plasmid SLI_NEO_PF3D7_1124300_AID_GFP_GlmS | medium supplemented with 400 µg/ml Geneticin G-418 Sulpate |
| *P. falciparum* 3D7 PF3D7_1128000-AID-GFP | *P. falciparum* 3D7 after transfection and confirmed genomic integration of plasmid SLI_NEO_PF3D7_1128000_AID_GFP_GlmS | medium supplemented with 400 µg/ml Geneticin G-418 Sulpate |
| *P. falciparum* 3D7 PF3D7_1212900-3xHA BDP2 | *P. falciparum* 3D7 after transfection and confirmed genomic integration of plasmid SLI_NEO_PF3D7_1212900_3xHA_GlmS BDP2 | medium supplemented with 400 µg/ml Geneticin G-418 Sulpate |
| *P. falciparum* 3D7 PF3D7_1225200-AID-GFP | *P. falciparum* 3D7 after transfection and confirmed genomic integration of plasmid | medium supplemented with 400 µg/ml Geneticin G-418 Sulpate |

| | | |
|---|---|---|
| | SLI_NEO_PF3D7_1225200_AID_GFP_GlmS SANT | |
| *P. falciparum* 3D7 PF3D7_1234100-AID-GFP TAF1 | *P. falciparum* 3D7 after transfection and confirmed genomic integration of plasmid SLI_NEO_PF3D7_1234100_AID_GFP_GlmS TAF1 | medium supplemented with 400 µg/ml Geneticin G-418 Sulpate |
| *P. falciparum* 3D7 PF3D7_1433400-AID-GFP PHD2 | *P. falciparum* 3D7 after transfection and confirmed genomic integration of plasmid SLI_NEO_PF3D7_1433400_AID_GFP_GlmS PHD2 | medium supplemented with 400 µg/ml Geneticin G-418 Sulpate |
| *P. falciparum* 3D7 PF3D7_1451200-2xFKBP-GFP-2xFKBP + episomal pLyn-FRB-mCherry | *P. falciparum* 3D7 with the endogenous PF3D7_1451200 gene tagged with 2xFKBP-GFP-2xFKBP using SLI and the episomal-expressed plasma-membrane mislocalizer, from (2) | medium supplemented with 400 µg/ml Geneticin G-418 Sulpate (no BSD selection pressure to maintain the mislocalizer plasmid was used) |
| *P. falciparum* 3D7 PF3D7_1475600-AID-GFP BDP4 | *P. falciparum* 3D7 after transfection and confirmed genomic integration of plasmid SLI_NEO_PF3D7_1475600_AID_GFP_GlmS BDP4 | medium supplemented with 400 µg/ml Geneticin G-418 Sulpate |
| *P. falciparum* 3D7 BDP5/TAF1-2xFKBP-GFP-2xFKBP + episomal pLyn-FRB-mCherry | *P. falciparum* 3D7 with the endogenous bdp5/taf1 gene tagged with 2xFKBP-GFP-2xFKBP using SLI and the episomal-expressed plasma-membrane mislocalizer, from (2) | medium supplemented with 400 µg/ml Geneticin G-418 Sulpate (no BSD selection pressure to |

| | | |
|---|---|---|
| | | maintain the mislocalizer plasmid was used) |
| *P. falciparum* 3D7 BDP5/TAF1-2xFKBP-GFP-2xFKBP + episomal pLyn-FRB-T2A-mCherry | *P. falciparum* 3D7 with the endogenous bdp5/taf1 gene tagged with 2xFKBP-GFP-2xFKBP using SLI and the episomal-expressed plasma-membrane mislocalizer, from (2) | medium supplemented with 400 µg/ml Geneticin G-418 Sulpate (no BSD selection pressure to maintain the mislocalizer plasmid was used) |

*Bacterial strains*

| **Name** | **carries plasmid** | **plasmid elements** | **description** |
|---|---|---|---|
| STBL3_NEO_AID_GFP_GlmS | SLI_NEO_AID_GFP_GlmS | *PF3D7_0525000_3'CDS-AID-GFP-T2A-NeoR-GlmS-3'PbDT 5'cam-hDHFR-3'hrp23 ampR* | *2xfkbp* from pSLI-2×FKBP-GFP (2) was replaced by *AID* and a *GlmS* was inserted after the *Neo-R* stop codon (https://benchling.com/s/BuTp2ktn) |
| STBL3_PF3D7_0306100_AID_GFP_NEO | SLI_NEO_PF3D7_0306100_AID_GFP_GlmS | *PF3D7_0306100_3'CDS-AID-GFP-T2A-NeoR-GlmS-3'PbDT 5'cam-hDHFR-3'hrp23 ampR* | Homology box of SLI_NEO_AID_GFP_GlmS replaced by *PF3D7_0306100 3'CDS* without STOP codon |

| | | | |
|---|---|---|---|
| | | | (https://benchling.com/s/seq-LVVs1Q2JKzpFCaENXIYh) |
| STBL3_GCN5_AID_GFP_NEO | SLI_NEO_PF3D7_0823300_AID_GFP_GlmS GCN5 | *PF3D7_0823300_3'CDS-AID-GFP-T2A-NeoR-GlmS-3'PbDT 5'cam-hDHFR-3'hrp23 ampR* | Homology box of SLI_NEO_AID_GFP_GlmS replaced by *PF3D7_0823300 3'CDS* without STOP codon (https://benchling.com/s/eWmuO0Fd) |
| STBL3_PHD1_3xHA_NEO | SLI_NEO_PF3D7_1008100_3xHA_GlmS PHD1 | *PF3D7_1008100_3'CDS-3xHA-T2A-NeoR-GlmS-3'PbDT 5'cam-hDHFR-3'hrp23 ampR* | Homology box of SLI_NEO_AID_GFP_GlmS replaced by *PF3D7_1008100 3'CDS* without STOP codon ; *AID-linker-GFP* sequence replaced by *3xHA* (https://benchling.com/s/kBjTVgUS) |
| STBL3_BDP1_AID_GFP_NEO | SLI_NEO_PF3D7_1033700_AID_GFP_GlmS BDP1 | *PF3D7_1033700_3'CDS-AID-GFP-T2A-NeoR-* | Homology box of SLI_NEO_AID_GFP_GlmS replaced by |

| | | | |
|---|---|---|---|
| | | *GlmS-3'PbDT 5'cam-hDHFR-3'hrp23 ampR* | *PF3D7_1033700 3'CDS* without STOP codon (https://benchling.com/s/seq-879UfSrXto3jm6ZINS85) |
| STBL3_PF3D7_1124300_AID_GFP_NEO | SLI_NEO_PF3D7_1124300_AID_GFP_GlmS | *PF3D7_1124300_3'CDS-AID-GFP-T2A-NeoR-GlmS-3'PbDT 5'cam-hDHFR-3'hrp23 ampR* | Homology box of SLI_NEO_AID_GFP_GlmS replaced by *PF3D7_1124300 3'CDS* without STOP codon (https://benchling.com/s/vWUUmpYc) |
| STBL3_PF3D7_1128000_AID_GFP_NEO | SLI_NEO_PF3D7_1128000_AID_GFP_GlmS | *PF3D7_1128000_3'CDS-AID-GFP-T2A-NeoR-GlmS-3'PbDT 5'cam-hDHFR-3'hrp23 ampR* | Homology box of SLI_NEO_AID_GFP_GlmS replaced by *PF3D7_1128000 3'CDS* without STOP codon (https://benchling.com/s/seq-Gz3N6j8nd9rTgeFEUlJ1) |
| STBL3_BDP2_3xHA_NEO | SLI_NEO_PF3D7_1212900_3xHA_GlmS BDP2 | *PF3D7_1212900_3'CDS-3xHA-T2A-NeoR-GlmS-3'PbDT 5'cam-hDHFR-3'hrp23 ampR* | Homology box of SLI_NEO_AID_GFP_GlmS replaced by *PF3D7_1212900 3'CDS* |

| | | | |
|---|---|---|---|
| | | | without STOP codon ; *AID-linker-GFP* sequence replaced by *3xHA* (https://benchling.com/s/seq-kepRbrMEnbNKsJ0Rz2iJ) |
| STBL3_PF3D7_1225200_AID_GFP_NEO | SLI_NEO_PF3D7_1225200_AID_GFP_GlmS SANT | *PF3D7_1225200_3'CDS-AID-GFP-T2A-NeoR-GlmS-3'PbDT 5'cam-hDHFR-3'hrp23 ampR* | Homology box of SLI_NEO_AID_GFP_GlmS replaced by *PF3D7_1225200 3'CDS* without STOP codon (https://benchling.com/s/3A6vujZ3) |
| STBL3_PF3D7_1234100_AID_GFP_NEO TAF1 | SLI_NEO_PF3D7_1234100_AID_GFP_GlmS TAF1 | *PF3D7_1234100_3'CDS-AID-GFP-T2A-NeoR-GlmS-3'PbDT 5'cam-hDHFR-3'hrp23 ampR* | Homology box of SLI_NEO_AID_GFP_GlmS replaced by *PF3D7_1234100 3'CDS* without STOP codon (https://benchling.com/s/seq-f0ktqtbEWDwiAKpZQ2Bi) |

| | | | |
|---|---|---|---|
| STBL3_PF3D7_1433400_AID_GFP_NEO PHD2 | SLI_NEO_PF3D7_1433400_AID_GFP_GlmS PHD2 | *PF3D7_1433400_3'CDS-AID-GFP-T2A-NeoR-GlmS-3'PbDT 5'cam-hDHFR-3'hrp23 ampR* | Homology box of SLI_NEO_AID_GFP_GlmS replaced by *PF3D7_1433400 3'CDS* without STOP codon (https://benchling.com/s/QxgLcE5J) |
| STBL3_PF3D7_1475600_AID_GFP_NEO BDP4 | SLI_NEO_PF3D7_1475600_AID_GFP_GlmS BDP4 | *PF3D7_1475600_3'CDS-AID-GFP-T2A-NeoR-GlmS-3'PbDT 5'cam-hDHFR-3'hrp23 ampR* | Homology box of SLI_NEO_AID_GFP_GlmS replaced by *PF3D7_1475600 3'CDS* without STOP codon (https://benchling.com/s/seq-1EkQIfwqyBE7Js0wLERU) |

Bacteria were transformed using NZY+ medium (final of 9.66mg/ml NZ amine, 4.83mg/ml yeast extract, 0.08M NaCl, 12mM MgCl2, 12mM MgCl2, 19mM glucose), plated on agar plates with 0.1mg/ml ampicillin and cultured in standard LB medium with 0.1mg/ml ampicillin.

*Plasmid DNA cloning*

For IP-MS/MS experiments, endogenous proteins were C-terminally tagged with a GFP or triple-HA tag using the selection-linked integration (SLI) system (2). In addition to a GFP- or HA-'fishing' moiety, plasmids were constructed to include two options for conditional knock-down to allow optimal

flexibility. The self-cleaving GlmS ribozyme sequence for degradation of the mRNA (for both GFP- and 3xHA-tagged proteins) (3) and the auxin-inducible degron (AID) system for knockdown at the protein level (only for GFP-tagged proteins) (4). AID-GFP Dual tagging was used as default option, fusing a relatively large tag of ~ 57kDa to the protein of interest. For two proteins, PF3D7_1212900/BDP2 and PF3D7_1008100/PHD1, AID-GFP tagged integration lines could not be obtained. We suspect that the large protein tag might interfere with essential protein functions. Therefore, we choose to tag these proteins with a very small 3xHA epitope tag instead, adding a mass of ~5.7kDa to the protein of interest. This was successful for both proteins. Regrettably, neither the AID- or GlmS-based inducible knock-down were successful in these lines. “GFP tagged” line for PF3D7_1451200 was previously published (2) and is C-terminally fused to an 2xFKBP-GFP-2xFKBP sandwich-tag in parasites that also carry an episomal pLyn-FRB-mCherry plasmid for inducible knock-sideways mistargeting to the plasma membrane.

Plasmid pSLI-2×FKBP-GFP (2) containing an unrelated homology box (PF3D7_0525000 3’CDS) was modified to include the GlmS ribozyme sequence right after the *Neo-R* stop codon. Subsequently, the *aid* sequence was amplified from plasmid pG390 (4) using primers AID_for_AvrII_linker and AID_rev_MluI (see **Supplementary Table S5** for primer sequences and PCR conditions) which included a ‘Ala-Gly-Ala-Gly-Gly-Ala-Ala-Arg-Ala-Ala’- linker upstream of the AID protein sequence. *Aid* PCR product and pSLI-2×FKBP-GFP (2) plasmid were digested using AvrII and MluI restriction enzymes. Subsequently, the *aid* sequence was ligated into the pSLI-2×FKBP-GFP backbone resulting in plasmid SLI_NEO_AID_GFP_GlmS (https://benchling.com/s/BuTp2ktn). Correct amplification and ligation were verified by Sanger-sequencing using primers AID_for_AvrII_linker and AID_rev_MluI. To generate the plasmids for AID-GFP tagging of PF3D7_0306100, GCN5, BDP1, PF3D7_1124300, PF3D7_1128000, PF3D7_1225200, TAF1, PHD2 and BDP4, 3’CDS sequences excluding the STOP codon where PCR amplified from 3D7 wt gDNA using the CDS_for_NotI and CDS_rev_AvrII primers (primers and PCR conditions listed in **Supplementary Table S5)**. Subsequently, PCR products and SLI_NEO_AID_GFP_GlmS plasmid were digested using NotI and AvrII restriction enzymes, followed by

agarose gel separation and gel extraction of the SLI_NEO_AID_GFP_GlmS backbone. PCR amplified homology boxes were ligated into the SLI_NEO_AID_GFP_GlmS backbone resulting in the plasmids listed in the table “Bacterial strains” above. All plasmids were sequence verified for correct integration of the correct sequence by Sanger-sequencing using primers plasmid_SLI_for and plasmid_SLI_AID_rev (**Supplementary Table S5**). To generate the plasmids for 3xHA tagging of PHD1 and BDP2, 3’CDS sequences excluding the STOP codon where PCR amplified from 3D7 wt gDNA using the CDS_for_NotI and CDS_rev_AvrII primers listed in **Supplementary Table S5** and first cloned into plasmid SLI_NEO_AID_GFP_GlmS as described above to generate SLI_NEO_PF3D7_1008100_AID_GFP_GlmS and SLI_NEO_PF3D7_1212900_AID_GFP_GlmS, respectively. The *3xha* tag sequence was PCR amplified from plasmid pBcam-HA-LB using primers 3xHA_for_AvrII_NaeI and 3xHA_rev_SalI (see **Supplementary Table S5**), which included a ‘Ala-Gly’-linker upstream of the 3xHA protein sequence. *3xha* PCR product and SLI_NEO_PF3D7_1008100_AID_GFP_GlmS and SLI_NEO_PF3D7_1212900_AID_GFP_GlmS plasmids were digested using AvrII and SalI restriction enzymes, followed by agarose gel separation and gel extraction of the plasmid backbones. Subsequently, the *3xha* sequence was ligated into the plasmid backbones and correct amplification and ligation were verified by Sanger-sequencing using primer plasmid_SLI_rev.

The sequence encoding the 292 terminal amino acids of BDP5 was PCR amplified using primers BDP5for and BDP5rev (**Supplementary Table S5**) and cloned into pSLI-sandwich plasmid (2) using NotI and AvrII. For SLI-TGD, base pairs 4 to 801 of the *bdp5* gene were PCR amplified with primers BDP5-TGDfor and BDP5-TGDrev (**Supplementary Table S5**) and cloned into pSLI-TGD (2) using Gibson cloning. The correct sequence of cloned inserts was verified by sequencing.

The primers FRB-T2A for and T2A-mCherry rev were used to obtain a PCR fragment that was cloned via KpnI/SpeI into Lyn-FRB-mCherry resulting in the plasmid Lyn-FRB-T2A-mCherry.

*Parasite transfection and generation of integrated lines*

3D7 wt parasites were transfected following the procedure of Fidock and Wellems (5) using a BTX electroporation system. Wild type 3D7 *P. falciparum* cultures were synchronized by sorbitol treatment and/or Percoll gradient centrifugation and young ring stage infected red blood cells (RBCs) were pelleted by centrifugation to 100% hematocrit. 300 µl infected RBCs were mixed with 150 µl sterile cytomix (120 mM KCl, 0.15 mM $CaCl_2 \cdot 2H_2O$, 5mM $MgCl_2 \cdot 6H_2O$, 25 mM HEPES, 2 mM EGTA, 10mM $K_2HPO_4$, 10mM $KH_2PO_4$) containing 100 µg plasmid DNA in a 2mm electroporation cuvette (BTX, #45-0125). Directly after electroporation, iRBCs were resuspended in warm culture media with 2% hematocrit and transferred to a shaking, semi-automated 37 °C incubator. 12-24 Hours after transfection, selection for episomal transfectants was performed by addition of 2.6nM WR99210 (Jacobus Pharmaceutical Company, Inc.). Parasites were cultured in the presence of WR99210 until they reached > 10 % parasitemia. WR99210 positive cultures were frozen twice after which WR99210 was removed from the culture media. Subsequently, selection for integrated parasites was started by addition of 400 µg/ml Geneticin G-418 Sulpate (Geneticin Selective Antibiotic (G418 Sulpate) powder, Gibco, Thermo Fisher Scientific, cat# 11811031, dissolved to 50 mg/ml in MQ). Twice a week, blood smears were checked for parasites. Once blood smears became positive, parasites were frozen and genomic DNA was isolated (QIAamp DNA Blood Mini kit, cat# 51106) to check for integration. Integration PCRs were performed on transgenic line genomic DNA as well as wt 3D7 genomic DNA as a control using 3 reactions each: i) an integration PCR using primers '*upstream_for' and 'plasmid_SLI_AID_rev' (for AID-GFP taggings) or 'plasmid_SLI_rev' (for 3xHA taggings), yielding PCR product only upon correct integration ; ii) a wt contamination PCR check using primers '*upstream_for' and '*3UTR_rev' to test for the presence of (residual) wt locus ; iii) a positive control PCR using primers '*upstream_for' and '*CDS_rev_AvrII' amplifying both from wt and transgenic parasites (see **Supplementary Figure S4A** for PCR setup and **S4B** for line validation; for primer sequences and PCR conditions see **Supplementary Table S5**). Often, residual wt signal was still present in the transgenic lines soon after they the first parasites were detected. If residual wt signal was

present, genomic DNA extraction and integration PCRs were repeated once or more with two-week intervals until all wild type PCR signal disappeared (**Supplementary Figure S4B**).

For transfection of *P. falciparum* parasites in the Spielman lab 100 μg of purified plasmid DNA (QIAGEN) were transfected with an Amaxa system (Nucleofector II AAD-1001N, program U-033) as previously described (6). Transfectants were selected using 4 nM for WR99210 (Jacobus Pharmaceuticals). SLI was done as described (2) by placing the culture on 400 μg/ml G418 (Sigma) after the parasites carrying the episomal plasmid had been established. When parasites re-appeared after G418 selection, a PCR across the integration junctions and one verifying absence of the unmodified locus was conducted to assess correct integration

*Nuclei isolation and generation of native nuclear extract*

Mixed stage, asynchronous *P. falciparum* parasites were grown for nuclear collection as described in '*Plasmodium falciparum* culturing'. The nuclear collection protocol was modified from (7). Upon harvesting parasite cultures were immediately transferred to ice to freeze all processes and subsequently filtered over PlasmoPure filters (EuroProxima, Netherlands) to remove human white blood cells. RBCs were washed in PBS once and subsequently resuspended in PBS with Protease Inhibitor Cocktail (PI at 1:100, Roche, cat# 04693132001) to a maximum of 6.25% hematocrit. Saponin was added to 0.05% and cultures were left incubating on ice for maximum 15 minutes. Parasites and lysed RBCs were layered over a double sucrose gradient in cell lysis buffer (CLB, 10mM Tris pH8.0, 3mM $MgCl_2$, 0.2% NP-40, PI at 1:50; bottom layer CLB/0.25M sucrose, top layer CLB/0.1M sucrose) and nuclei were isolated by centrifugation (4°C, 12min, 3400 x g, acceleration and deceleration set at slow). Nuclei were resuspended in CLB with 20% glycerol, pelleted by centrifugation in Eppendorf tubes (4°C, 10min, 3500 x g, table-top centrifuge), snap-frozen and stored at -80°C until the generation of the nuclear extract. Nuclear extract was generated as in (8) as follows. Upon thawing, isolated nuclei were washed once with buffer (50mM Tris pH7.4, 4mM $MgCl_2$, 1mM $CaCl_2$, Protease Inhibitor Cocktail) and pelleted by centrifugation for 13 min at 1500 x g. Supernatant was removed and pellet

volume determined as accurately as possible. Nuclei were resuspended in 6 pellet volumes of High Salt Extraction Buffer (modified from (9); 50mM HEPES pH7.5, 20% glycerol, 420mM NaCl, 1.5mM MgCl2, 1mM DTT, 0.4% NP40, PI with optional 400 units/ml DNaseI (Roche, cat# 284932001)), nuclear membranes ruptured by 15-20 strokes of an Eppendorf-douncer and proteins extracted by rotation for 2 hours at 4 °C. Subsequently, samples were centrifuged for 10min at 17000 x g and nuclear extract was collected. To enhance efficiency of protein extraction a second extraction was performed on the pelleted nuclei using 4 original-pellet volumes of High Salt Extraction Buffer, 2 hours rotation at 4 °C followed by spinning for 10min at 17000 x g. The second extract was collected and pooled with the first extract, after which nuclear extracts were quantified using a Qubit fluorometer (Qubit™ Protein Assay Kit, Thermo Fisher Scientific, #Q33212), aliquoted, snap-frozen and stored at -80 °C. For histone peptide experiments nuclear extracts were generated without addition of DNaseI to reduce the amount of free histones in the nuclear extract. For GFP and HA-IP experiments, DNaseI was included in the nuclear extract to increase the extraction of (hetero)chromatin associated proteins.

*Quantitative Histone Peptide pulldown*

The histone peptide pulldown protocol was modified from (10,11) and protocols made available by Cellzome. Most experiments were designed to contain 3 dimethyl labels (“light”, “medium”, “heavy”) and included two modified histone peptide reactions and a common unmodified control peptide reaction for quantitative analysis. The unmodified control peptide was of the exact same sequence as the modified peptide(s), but lacked the specified PTMs. Some experiments - listed below - contained only a single modified peptide reaction and one unmodified control peptide reaction and only used a “light”- and “heavy”- dimethyl label for quantitative analysis (H2B.Z_5ac exp 17 and 18 ; H3K4me1 exp 1 and 2 ; H4_3ac exp 5, 6, 7, 8, 13, 14, 15 and 16 ; H4K20me3 exp 1, 2, 3 and 4). Used peptides are listed in the table below. Technical replicates were included for each nuclear extract with subsequent numbering (e.g. experiment 1 and 2 are always technical replicates) and were performed using label-

swap conditions for di-methyl labelling (uneven numbered experiments were performed as forward reactions, even numbered experiments were reverse reactions). For each peptide, except H3_2ac (K9acK14ac), multiple biological replicates were performed from independent nuclear extracts.

Directly before pull-down, nuclear extracts were thaw and diluted to the following buffer composition (50mM HEPES pH7.5, 10% glycerol, 350mM NaCl, 1.5mM MgCl2, 1mM DTT, 0.4% NP40, PI, 200nM Trichostatin A (TSA, Sigma-Aldrich cat# T8552, dissolved to 5mM in DMSO and further diluted to a 200µM stock in mass-spec grade MQ, stored at -20°C)) and a 0.88 mg/ml protein concentration. Precipitations were removed by centrifugation (25min, 17000 x g, 4°C) and transfer of the supernatant to new tubes. 15 µl of beads (Streptavidin-Sepharose High Performance beads, GE Healthcare cat# 17-5113-01, 50% slurry so use 30µl of beads slurry per rxn) were used per reaction and were washed once with 1ml buffer (150mM NaCl, 50mM HEPES pH8.0, 0.1% NP40 ; spin 2min, 400 x g, 4 °C) prior to peptide binding. 2.63nmol of biotinylated peptide was allowed to bind the beads while rotating for at least 30min at 4°C in 0.5ml of buffer volume (150mM NaCl, 50mM HEPES pH8.0, 0.1% NP40). Unbound peptide was removed by three washes with 1ml buffer (350mM NaCl, 50mM HEPES pH8.0, 1% NP40, 10µM ZnCl2, 0.5mM DTT; spin 2min, 400 x g, 4 °C). Subsequently, 575µg of nuclear extract – unless indicated otherwise - was added to the beads and histone reader complexes were allowed to bind for 2 - 3 hours while rotating at 4°C in a 650µl volume. Subsequently, beads were washed 3 times with 1ml wash buffer 1 (350mM NaCl, 50mM HEPES pH8.0, 1% NP40, 10µM ZnCl2, 0.5mM DTT, 200nM TSA, PI), 2 times with 1ml wash buffer 2 (350mM NaCl, 50mM HEPES pH8.0, 10µM ZnCl2, 0.5mM DTT, 200nM TSA) and once with wash buffer 3 (350mM NaCl, 100mM Triethylammonium bicarbonate (TEAB, 1.0M stock pH8.5, Sigma-Aldrich, #T7408-100ml, diluted to 100mM in mass-spec grade MQ and stored at RT), 10µM ZnCl2, 0.5mM DTT, 200nM TSA). After the final wash, the supernatant was removed as much as possible and on-bead digestion was performed as in (12) as described below. 2.5µl of Tris(2-carboethyl)phosphine hydrochloride (TCEP, Sigma, #C4706-2G, dissolved to 100mM in mass-spec grade MQ and stored at -20 °C) was added to a final concentration of 5mM and disulfide bonds were reduced for 1 hour while shaking in a thermoshaker at 37°C to prevent settling of the

beads. Cysteine alkylation was induced by addition of 2.5µl methyl methanethiolsulfonate (MMTS, Thermo Scientific, #23011, dissolved to 200mM in 100% isopropanol and stored at -20°C) to a final concentration of 10mM followed by a 10 min incubation in a thermoshaker at 37°C. Proteins were subsequently digested off the beads by addition of 0.4µg Trypsin/LysC (Promega, #V5072, dissolved to 0.4ug/µl in mass-spec grade MQ and stored at -20°C) and 1 hour incubation while shaking in a thermoshaker at 37°C. Beads were pelleted by 2 min centrifugation at 400 x g at RT and 53.5µl of supernatant was transferred to a clean Eppendorf tube. 50µl of 100mM TEAB was added to the beads followed by a 5 min incubation while shaking at 37°C, after which the beads were again pelleted by 2 min centrifugation at 400 x g at RT. 50µl of supernatant was removed from the beads and pooled with the first supernatant to improve the efficiency of protein isolation. Protein digestion was continued O/N (~16-19 hours) in a 37°C waterbath. Di-methyl labelling was performed as in (13) as follows. 4µl of a 4% label ("light": $CH_2O$, 37% formaldehyde solution, Sigma-Aldrich #252549-25ml ; "medium": $CD_2O$, 20% formaldehyde, d2 solution, Sigma-Aldrich #492620-20G ; "heavy": $^{13}CD_2O$, 20% formaldehyde-$^{13}C$, d2 solution, Sigma-Aldrich #596388-20G, diluted to 4% in mass-spec grade MQ prior to use) was added to each 103.5µl sample, followed by addition of 4µl 0.6M $NaBH_3CN$ (Merck, #818053, freshly dissolved in mass-spec grade MQ immediately prior to use) to the "light" and "medium" or 4µl of 0.6M $NaBD_3CN$ (Sigma, #190020-1G, freshly dissolved in mass-spec grade MQ immediately prior to use) to the "heavy" reactions. Samples were incubated for 1 hour while shaking at RT, after which the reaction was stopped by addition of 16µl 1% ammonia. For each experiment "light"-, "medium"- and "heavy"-labelled samples were pooled into a single tube and the sample pool was acidified by addition of 15µl 100% trifluoroacetic acid (TFA, Biosolve BV, #20234131, stored at RT). Each sample-pool of 3 labels was cleaned and concentrated divided over two 3-disk-C18 stage-tips (14), while pools of two labels only were cleaned and concentrated over a single 3-disk-C18 stage-tip. Loaded stage-tips were stored at 4°C until loading for mass spectrometry analysis.

Peptides were ordered with a C-terminal biotin from Alta Bioscience either as off-the-shelf items (corresponding to human peptide sequence, if available) or generated as custom orders. Peptides were required to have a minimal purity of 90% with no more than 5% purity difference between modified and corresponding control peptide.

H3_2ac, H3K9me3, H3K4me3 (short) and corresponding H3_unmod (short) control peptides were previously reported in (11) and were a kind gift of dr. Nina Hubner and Michiel Vermeulen.

| **Peptide name** | **Sequence** | **Stock conc** | **Solvent** | **Purity** | **Source** |
|---|---|---|---|---|---|
| H2A.Z_unmod | MEVPGKVIGGKVGGKVGGKVLGLGKGGKGKTGSGKTKKA-GG-K(biotin)-amide | 0.5mM | 0.1% acetic acid | 83.2% | Alta Bioscience cat# M2847B $ |
| H2A.Z_4ac | MEVPGKVIGGKVGGKVGGKVLGLGK(Ac)GGK(Ac)GK(Ac)TGSGK(Ac)TKKA-GG-K(biotin)-amide | 0.5mM | 0.1% acetic acid | 85.4% | Alta Bioscience cat# M2847C $ |
| H2A.Z_7ac | MEVPGKVIGGK(Ac)VGGK(Ac)VGGK(Ac)VLGLGK(Ac)GGK(Ac)GK(Ac)TGSGK(Ac)TKKA-GG-K(biotin)-amide | 0.5mM | 0.1% acetic acid | 81.8% | Alta Bioscience cat# M2847D $ |
| H2B.Z_unmod | SGKGPAQKSQAAKKTAGKTLGPRH-GG-K(biotin)-amide | 0.5mM | 0.1% acetic acid | 83.3% | M2828A $ |

| | | | | | |
|---|---|---|---|---|---|
| H2B.Z_2ac | SGK(Ac)GPAQK(Ac)SQAAKKTAGKTLGPRH-GG-K(biotin)-amide | 0.5mM | 0.1% acetic acid | 83.3% | Alta Bioscience cat# M2847A $ |
| H2B.Z_3ac | SGKGPAQKSQAAK(Ac)K(Ac)TAGK(Ac)TLGPRH-GG-K(biotin)-amide | 0.5mM | 0.1% acetic acid | 81.9% | Alta Bioscience cat# M2828B $ |
| H2B.Z_5ac | SGK(Ac)GPAQK(Ac)SQAAK(Ac)K(Ac)TAGK(Ac)TLGPRH-GG-K(biotin)-amide | 0.5mM | 0.1% acetic acid | 85.4% | Alta Bioscience cat# M2828C $ |
| H3_unmod (short) | ARTKQTARKSTGGKAPRKQ-GG-W-K(biotin) ^ | 0.4mM | Buffer & | - | (11) |
| H3_unmod (long) | ARTKQTARKSTGGKAPRKQLA-Ahx-K(biotin)-amide ^ | 0.5mM | 0.1% acetic acid | 96.6% | Alta Bioscience cat# HS27 B#M2862C |
| H3K4me1 | ARTK(Me1)QTARKSTGGKAPRKQLA-Ahx-K(biotin)-amide ^ | 0.5mM | 0.1% acetic acid | 93.8% | Alta Bioscience cat# M2888 $ |

| | | | | | |
|---|---|---|---|---|---|
| H3K4me2 | ARTK(Me2)QTARKSTGGKAPRKQLA-Ahx-K(biotin)-amide ^ | 0.5mM | 0.1% acetic acid | 95.1% | Alta Bioscience cat# HS04 B#M2197A1 |
| H3K4me3 (short) | ARTK(Me3)QTARKSTGGKAPRKQ-GG-W-K(biotin) ^ ! | 0.4mM | Buffer & | - | (11) |
| H3K4me3 (long) | ARTK(Me3)QTARKSTGGKAPRKQLA-Ahx-K(biotin)-amide ^ | 0.5mM | 0.1% acetic acid | 98.2% | Alta Bioscience cat# HS60 B#M2705B |
| H3_2ac | ARTKQTARK(Ac)STGGK(Ac)APRKQ-GG-W-K(biotin) ^ | 0.4mM | Buffer & | - | (11) |
| H3K9me3 | ARTKQTARK(Me3)STGGKAPRKQ-GG-W-K(biotin) ^ | 0.4mM | Buffer & | - | (11) |
| H4_unmod (short) | SGRGKGGKGLGKGGAKRHRKV-Ahx-K(biotin)-amide * | 0.5mM | 0.1% acetic acid | >95% | Alta Bioscience cat# M2686A1 |
| H4_unmod (long) | SGRGKGGKGLGKGGAKRHRKILRDN-GG-K(biotin)-amide | 0.5mM | 0.1% acetic acid | 87.9% | Alta Bioscience cat# M2862B $ |

| | | | | | |
|---|---|---|---|---|---|
| H4_3ac | SGRGK(ac)GGK(ac)GLGK(ac)GGAKRHRKV-Ahx-K(biotin)-amide * | 0.5mM | 0.1% acetic acid | 95% | Alta Bioscience cat# M2686A2 |
| H4K20me3 | SGRGKGGKGLGKGGAKRHRK(Me3)ILRDN-GG-K(biotin)-amide | 0.5mM | 0.1% acetic acid | 90.4% | Alta Bioscience cat# M2862A $ |

^ All H3 peptides were ordered off-the-shelf at Alta Bioscience as human histone H3 or provided by Dr Nina Hubner and Dr. Michiel Vermeulen and correspond to *P. falciparum* H3.3 histone sequence which differs at position 12 for the *P. falciparum* H3 histone tail (H3 A12, H3.3 G12)

* The H4_3ac and control peptides were ordered off-the-shelf corresponding to the human histone H4 tail sequence which differs on the last amino acid from the *Plasmodium* sequence (human V21, P. falciparum I21)

& Buffer composition (150mM NaCl, 50mM HEPES pH8.0, 0.1% NP40)

$ custom ordered peptides

! In H3K4me3 exp 1, 2, 7 and 8 instead of the peptide carrying solely a H3K4me3 modification, the multi-modified H3K4me3K9acK14ac peptide (11) was used. Since the addition of H3K9acK14ac had no effect on the recruited target proteins, we decided to analyze these experiments as being H3K4me3. These experiments increased our confidence that in *P. falciparum*, under this experimental setup, H3K9acK14ac by itself is not sufficient to recruit reader complexes nor does it contribute to recruitment by the H3K4me3 mark. We therefore refrained from performing additional replicates with the H3_2ac peptide.

For some experiments, the setup differed slightly from the standard setup described above. Deviations are listed below:

Some experiments were performed with deviating amounts of nuclear extract as input for the modified as well as the control peptide control reactions. These are H2A.Z_4ac exp 3 and 4 (1mg input), H2A.Z_7ac exp 3 and 4 (1mg input) ; H4_3ac exp 9, 10, 11, 12, 13 and 14 (250µg input) ; H4_3ac exp 15 and 16 (750µg input).

For the H3K4me3 pulldowns several different peptides were used. H3K4me3 experiments 3 – 6, 13 and 14 were performed using the H3K4me3 (short) peptide (11) covering the first 19 amino acids of the *P. falciparum* H3.3. tail sequence, while experiments 1, 2, 7 and 8 were performed with the same peptide sequence which in addition to H3K4me3 also carried acetylation on K9 and K14 (H3K4me3K9acK14ac (11)). Experiments 9 – 12 were performed using the longer H3K4me3 peptides obtained from Alta Bioscience that covered the first 21 amino acids of the *P. falciparum* H3.3. tail sequence. When quantitative comparisons amongst H3K4me1, H3K4me2 and H3K4me3 were made, these always dependent on experiments using the long H3 peptides. Since the addition of H3K9acK14ac to the H3K4me3 peptide had no effect on the recruited target proteins, nor reveal additional interactors, we decided to analyze these experiments as being H3K4me3. Importantly, these experiments increased our confidence that in *P. falciparum*, under this experimental setup, H3K9acK14ac by itself is not sufficient to recruit reader complexes nor does it contribute to recruitment by the H3K4me3 mark.

H2A.Z_7ac exp 7 and 8, H2B.Z_5ac exp 1, 8, 11 and 12 and H4_3ac exp 1 and 2 contained the general bromo-domain inhibitor bromosporine in the acetylated-peptide reaction prior to and during the peptide incubation step, while in H4_3ac exp 10 and 11 an acetyl-lysine competitor peptide was present in these steps of the procedure. Since neither of these competitors had any effect on reader-binding, these reactions were included in the analysis and heatmap in **Figure 1C**.

*Recombinant PHD1 PHD-domain peptide binding assay*

The fourth PfPHD1 PHD domain was expressed in *E.coli* as a GST-fusion protein. Briefly, a gene fragment coding the fourth PHD domain of *Pf*PHD1 (nucleotides 11374-11562, amino acids 3792-3853) was amplified using primer PHD-domain for (with BamHI site) and PHD-domain rev (with XhoI site) and cloned into pGEX-6P-1 vector. Soluble GST-fusion protein was purified by glutathione sepharose 4b (GE Healthcare) based on the manufacture's protocol. The peptide binding assay was conducted as described earlier by Chang and colleagues (15). Briefly, purified GST-PHD was incubated with biotinylated H3 peptides in the presence of streptavidin Sepharose resin (GE Healthcare). The beads were collected via centrifugation and washed five times with peptide binding buffer (50 mM Tris, pH 7.5, 150 mM NaCl, and 0.05% Nonidet P-40). Bound protein was detected by Western blot using anti-GST antibodies. H3 peptides include unmodified H3 and singly modified H3K4me1/2/3 H3 peptides (corresponding to amino acids 1–21 of the H3 histone tail, see peptide table).

*Quantitative GFP- and HA-pulldowns*

The GFP- and HA-pulldown procedures were modified from (16) and were performed as follows. Experiments were setup as technical replicates, which were number sequentially (e.g. forward reactions contain uneven numbers, reverse reactions are even numbered). Biological replicate experiments from independent nuclear extract were also performed for each bait. For each experiment a GFP- or HA-binding reaction was performed in parallel to a negative control pulldown on the same input material. Technical replicates were performed under label-swap conditions using "light" and "heavy" di-methyl labelling. Quantitative proteomics was performed to distinguish proteins binding significantly to the GFP- or HA-beads over background proteins. In order to exclude false-positive proteins that are not part of the tagged-proteins complex, but show direct enhanced binding to the GFP-Trap of HA-antibody beads, two negative controls were included. 1. A HA-negative control in which HA-bead and control bead pulldowns were performed from a nuclear extract from

GFP-tagged parasites; and 2. a GFP-negative control in which GFP-Trap and control bead pulldowns were performed on a wild-type (exp 3 and 4) or HA-tagged (exp 1 and 2) nuclear extract.

Directly prior to the pulldown, nuclear extracts were thaw and diluted to the following buffer composition (50mM HEPES pH7.5, 300mM NaCl, 18.2% glycerol, 1.5mM MgCl2, 0.2mM EDTA, 0.4% NP40, 1mM DTT, PI, 50µg/ml ethidium bromide) and a minimum protein concentration of 2mg/ml. Precipitations were removed by spinning (25min, 17000 x g, 4°C) and transferred to a clean tube. For GFP-pulldowns, 7.5µl of GFP-binding beads (GFP-Trap®_A, Chromotek cat# gta-20, use 15µl of 50% slurry per rxn) or 7.5µl of negative control beads (Blocked Agarose Beads (BAB), Chromotek cat# bab-20, use 15µl of 50% slurry per rxn) per reaction were used. For HA-pulldowns 10µl of HA-binding beads (HA-Tag, C29F4 Rabbit mAb Sepharose Bead conjugate, Cell Signalling cat# 3956S, use 20µl of 50% slurry per reaction) or 10µl of negative control beads (Rabbit (DA1E) mAb IgG XP Isotype control Sepharose Bead conjugate, Cell Signalling cat# 3423, use 20µl of 50% slurry per reaction) were used. Beads were washed 3 times with 1ml buffer (300mM NaCl, 50mM HEPES pH7.5, 18.2% glycerol, 1.5mM MgCl2, 0.2mM EDTA, 0.4% NP40, 1mM DTT, PI) after which 500µl of nuclear extract (1 – 2.6 mg) was added to the washed beads. Protein capture was allowed by incubation for 90min while rotating at 4°C. Subsequently, beads were washed twice with 1ml wash buffer (300mM NaCl, 50mM HEPES pH7.5, 18.2% glycerol, 1.5mM MgCl2, 0.2mM EDTA, 0.5% NP40, 1mM DTT, PI), twice with 1ml (PBS, 0.5% NP40), twice with 1ml PBS and once with 1ml 100mM TEAB buffer. All procedure steps were performed at 4°C unless indicated otherwise and all wash spinning steps for 2min 400 x g. After the final wash, the supernatant was removed as much as possible and on-bead digestion and di-methyl labelling were performed as described in "*Quantitative Histone Peptide pulldown*" using only "light" and "heavy" di-methyl labels. GFP- or HA-IP and control reactions were pooled and each sample pool was cleaned and concentrated over 5-disk-C18 stage-tips (14). When <1.5mg input was used per reaction the sample pool was loaded on a single stage-tip. When >1.5mg input was used the pool was split over two stage-tips. Loaded stage-tips were stored at 4°C until loading for mass spectrometry analysis.

*Microscopy*

Fluorescence microscopy was carried out essentially as described (17). Briefly, 1 µg/µl DAPI was added to the parasites in culture medium and incubated for 15 min at room temperature. For imaging a drop of this suspension was placed on a glass slide and covered with a coverslip. Images were acquired with a Zeiss AxioImager M1 or M2 microscope with a Hamamatsu Orca C4742-95 camera controlled by AxioVision software. A 63x plan-apochromate oil immersion objectives with an aperture of 1.4 was used for all images. Brightness and intensity of images adjustments and overlays were done using Corel Photo Paint (version X6).

To assess the efficiency of the BDP5 knock sideways Fiji was used to draw a cross section through cells in fluorescence microscopy images of live parasites in a way that it traversed the nucleus using synchronised DAPI and GFP channel images. The 'plot profile' function was used to measure the fluorescence intensity along the drawn cross-section.

*Mass spectrometry*

For each Histone Peptide Pulldown sample pool, a single 3xC18 disk stage-tip was eluted. For each GFP- or HA-pulldown sample pool either the single (when inputting <1.5mg NE per rxn) or both (when inputting >1.5mg NE as input per rxn) 5xC18-disk stage-tips were eluted. Loaded stage-tips that were stored at 4°C for prolonged periods were rehydrated with 25µl buffer A (0.1% formic acid) prior to elution. Stage-tips that were loaded directly before elution omitted the rehydration step. Peptides were eluted using 30µl buffer B (80% acetonitrile, 0.1% formic acid) per stage-tip in PCR-tubes. Where applicable, multiple stage-tips of the same sample pool were pooled in a single PCR-tube. Acetonitrile evaporation was achieved by 15-35 min vacuum spin at room temperature to ~5-8µl volume, after which each sample was reconstituted to 12µl with buffer A. 10µl of reconstituted sample (~83% of total sample) for GFP- of HA-pulldowns or 5µl of reconstituted sample (~21% of total sample-pool when pooling 3 labels, ~42% of total sample-pool when pooling 2 labels) for HPP experiments was

separated over a 30cm C18-reverse phase column (1.8µm Reprosil-Pur C18-AQ, dr. Maisch 9852) and eluted using an Easy-nLC 1000 (Thermo Fisher Scientific) over a 94min gradient (5.6% acetonitrile/0.1% formic acid - 25.6% acetonitrile/0.1% formic acid). Eluted peptides were directly injected into a QExactive mass spectrometer (Thermo Scientific). Data was acquired in TOP10 data-dependent acquisition mode with dynamic exclusion enabled for 20sec. Resolution for MS was set at 70.000 at m/z = 400 and for MS/MS at 17.5000.

*MS data processing*

Raw mass spectra were analyzed similar to (8), as follows. All histone peptide pulldown MS raw files were combined in a single analysis run to increase protein identification and comparability between individual pulldowns. For the same reason all GFP- and HA-pulldown MS raw files that are displayed together in a single figure were combined into one analysis. Xcalibur raw files were processes using MaxQuant (version 1.5.3.30 (18)) set to default parameters unless indicated. Multiplicity was set at 2 for GFP- and HA-pulldowns, with an added mass of 28.03Da ("light"-) or 36.08Da ("heavy"-label) to all lysine residues and peptide N-termini. Multiplicity was set at 3 for most histone peptide pulldowns (except those were only a "light" and a "heavy" label were pooled), with an added mass of 28.03Da ("light"-), 32.06Da ("medium"-) or 36.08Da ("heavy"-label) to all lysine residues and peptide N-termini. Trypsin/P was set as the specific digestion mode with maximum 2 missed cleavages and a mass of 45.99Da (MMTS) was set as fixed modification of cysteine residues. Match-between-runs and re-quantify options were enabled with default parameters and iBAQ values were calculated. Mass spectra were compared to peptide masses from the *Plasmodium falciparum* 3D7 annotated proteome (PlasmoDB release 33) with the entire human proteome included in the contaminants list using the integrated Andromeda search engine. For the GFP- and HA-pulldowns the protein sequence of the AID-GFP tag was added to the proteome to enable identification of the tag and use enrichment of the tag as a positive control for technical performance. Default search settings (mass tolerance at 4.5 ppm

for precursor ions and 20 ppm for fragment ions) were enabled, and peptides and proteins were accepted with an 0.01 FDR cut-off. Protein quantification required minimally a single “unique + razor” peptide-ratio to increase assay sensitivity. However, since single-peptide identifications are more prone to variation, more stringent filtering for at least 2 peptides has been employed in most downstream analysis (as indicated).

The MaxQuant output ProteinGroup file was further analyzed using the Perseus software package (version 1.4.0.20 (19)). Sample pools containing two labels only (GFP- and HA-pulldowns, some Histone Peptide pulldowns) output a single H/L ratio, whereas sample pools consisting of three labels (most Histone Peptide pulldowns) output M/L, H/L and H/M ratios. M/L, H/L and H/M (normalized) ratios were log2 transformed while intensity values were log10 transformed. After filtering on ‘only identified by site’, ‘reverse’ and ‘potential contaminant’ hits the Histone Peptide pulldown dataset retained 1368 of the original 1668 protein identifications, the GCN5/PHD1/PHD2/negative controls GFP- and HA-pulldown dataset (**Figure 3**) retained 516 out of 1473 of the original protein identifications, whereas for the BDP1/BDP2/BDP4/PF3D7_0306100/PF3D7_1124300/PF3D7_1128000/PF3D7_1225200/PF3D7_1451200/TAF1/negative_controls GFP- and HA-pulldown dataset (**Figure 4**) 709 of the original 1593 proteins remained. Significant-outliers were determined using the intensity-based Significance B option (two-sided Benjamini-Hochberg test) with an FDR cut-off set to 0.05. A –x transformation was applied to M/L, H/L or H/M (normalized) ratios where necessary to obtain experiment/control ratios (e.g. GFP-Trap/BAB control, HA/IgG control bead or modified peptide/unmodified peptide). Further processing of the Histone Peptide pulldown and GFP-/HA-pulldown files was performed in excel.

*Histone Peptide pulldown analysis*

Further processing of the Perseus output file to **Supplementary Table S2** was performed in excel.

Scatterplots of replicate experiments were generated in R (R i386 3.3.3 (20)). Only normalized log2-ratios of protein identifications based on at least two peptides of which minimally one unique were included in the plots and significantly enriched (red) or depleted (blue) outliers with an FDR <5% in both experiments were depicted as diamonds and labelled with the protein name. PF3D7_1140700 (**Figure 1B & S1A**, H3K9me3 panel – transparent red) has been manually added by exception, as it did not pass the stringent selection criteria of at least 2 peptides per experiment. PF3D7_1140700 has only been identified with a single peptide in H3K9me3 experiment 1 and 2, thus has been added to the plot based on its single peptide normalized log2-ratios. Considering it is a solid – though lowly identified – significantly enriched protein hit in most H3K9me3 pulldowns (sometimes it was identified but a NaN ratio was given due to lack of signal for the control label) we believe it deserves highlighting in **Figure 1B & S1A**. For optimal visualization, the axes of scatterplots in **Figure 1B, Figure 2 and Figure 3A** have been manually scaled, centered around the (0,0) axis point to include all significantly enriched as well as background proteins. Using this scaling, significantly depleted proteins are not all present in the H3K4me3 and H3K9acK14ac plots in **Figure 1B**, or in the H4_3ac plot of **Figure 2A**. Full, auto-scaled scatterplots of these experiments are included in **Supplementary Figure S1, S2 and S3**. In the main Supplementary Figure catterplots, we omitted significantly repelled proteins that fell outside of the plotted area as we consider significantly depleted proteins likely false-positives for the following reasons: i) significantly repelled proteins often included chaperones and ribosomal proteins which are known to be common contaminants in proteomic experiments, ii) histones were giving very variable results and were often found as either significantly depleted or significantly enriched which would suggest that they are likely to provide false-positive hits on either side of the spectrum in HPP experiments, iii) significantly repelled proteins were often consistent when using the same nuclear extract, but varied when using different nuclear extract. This is in contrast to significantly enriched proteins which were enriched in multiple biological replicate experiments, iv) HPP experiments performed in other organisms reliably reveal readers recruited to particular histone PTMs but fail to identify true repulsion of proteins (personal communication with various participants of FEBS Nuclear

Proteomics Lecture Course 2014), which is in line with the lack of consistent repelled protein hits we observe in our *P. falciparum* experiments. Raw scatterplots were further adjusted in Adobe Illustrator.

The heatmaps in **Figures 1C** & **S3B** only contains proteins that were identified by two peptides or more in the HPP dataset and were considered significantly recruited for one or more PTMs. Significant recruitment was defined as consistent binding to a certain histone PTM. In order to include potential stage-specific or substochiometric complex components, but as much as possible reduce the noise-level we choose to only include proteins in the heatmap that were significantly enriched (FDR <5%) in more than 20% of the experiments for a certain PTM (e.g. significant in at least 4 out of 18 experiments for H4_3ac) with a minimum set at 2 reactions (e.g. significant in at least 2 out of 4 experiments for H2B.Z_3ac). Ribosomal proteins and histones were considered to be false-positives (as they yielded very inconsistent results, often being significant on the enriched or on the depleted side of the spectrum) and were therefore removed from the heatmaps. The background cloud in normalized log2-ratio histone peptide pulldowns was often shifted from the (0,0) center, in particular for the pulldowns with highly acetylated histone peptides which often bound less overall protein likely due to their reduced charged state in the incubation and wash buffers. Seemingly the MaxQuant algorithm does not succeed in proper normalization of the raw ratios, perhaps because it also includes contaminating human peptides to determine the normalization factor. As absolute normalized log2-ratios are therefore less informative and should always be considered respective to the location of the background cloud in each individual experiment, we choose to display FDR values in the heatmap of figure 1A rather than using absolute normalized log2-ratios. For Supplementary Figure S3B we display both heatmaps of methylated H3k4 peptides based on enriched FDR values as well as normalized log2 PTM-over-unmodified peptide control ratio's. The heatmaps were generated using the web-based Morpheus tool from the Broad Institute (21), plotting the FDR for enrichment (Benjamini-Hochberg Significance B). Proteins depleted compared to the median normalized log2-ratio for each experiment obtained an FDR of 1.  NaN values - where either a protein is not identified in an experiment or no ratio can be given due to a missing label - are depicted as white/grey blocks in the heatmap. A 4-colour

scale was employed: dark red for very significant binders (FDR 0), bright red on the significance cut-off (FDR 0.05), light yellow for sub-significantly recruited proteins (FDR 0.1) and blue for non-significant binding (FDR 1). The normalized log2 PTM-over unmodified peptide ratio-based heatmaps used a 3-colour scale (blue= depleted, yellow= neutral, red= enriched using the 1 to 99 percentile range of all log2 normalized ratios present in the H3K4me1, me2 and me3 combined dataset) with NaN values highlighted as grey blocks. For the heatmap in figure 1C, hierarchical clustering (HC) on the columns (experiments) was performed using a 2-layered approach. First, histone PTMs were hierarchically clustered on their average FDR value. The dendrogram for this clustering is included in the figure and determines the order in which the peptides are listed. Second, for each peptide, the order of experiments was based on a hierarchical clustering within each class. For the rows (identified proteins), an initial k-means clustering into 4 clusters was followed by a HC-based sub-clustering within each k-means group. Four clusters were chosen as that seemed to best capture the structure of the dataset. Clusters are indicated in blocks with different shades of grey between heatmap and protein IDs. For the heatmap in **Supplementary Figure S3B** clustering was performed on the FDR-based heatmap using the same two-step HC as in **Figure 1C** for the columns, but a single HC-based clustering for the rows. Hierarchical clustering and k-means clustering were performed using the integrated clustering algorithm of Morpheus set at default parameters (for HC using the 'one minus Pearson' correlation and 'average linking' method, for k-means using the 'one minus Pearson' correlation, 4 clusters and maximum 1000 iterations). PlasmoDB gene IDs are listed for the identified proteins, with abbreviated names included between brackets where possible. Red bolded proteins were selected for tagging and follow-up by GFP- and HA-IP-MS/MS, underlined proteins were considered expected chromatin- or transcription-related proteins based on name or domain presence. Proteins that the authors would consider likely contaminants based on protein name, proven localization or domain presence are listed in grey.

*GFP- and HA-pulldown analysis*

Further processing of the Perseus output file to **Supplementary Table S3** and **S4** was performed in excel.

Scatterplots of technical replicates (**Supplementary Figure S4**) were generated in R (R i386 3.3.3 (20)) and were adjusted using Adobe Illustrator. Only normalized log2-ratios of protein identifications based on at least two peptides of which minimally one unique were included in the plots and significantly enriched (red) or depleted (blue) outliers with an FDR <10% in both experiments were depicted as diamonds and labelled with the protein name. Proteins significantly enriched (see **Supplementary Table S3**) or depleted in GFP- and HA-control experiments were considered false-positives and were removed from the plots. For optimal comparability, scatterplots have been scaled to identical axis for GCN5gfp, PHD1ha and PHD2gfp experiments. The SAGA-like components PHD1, PHD2 and PF3D7_1402800 are highlighted black when identified, but non-significant. Baits used for fishing are indicated in **bold** *italized* fond.

For the heatmaps in **Figures 3D** and **4A** only proteins that were identified by minimally two unique peptides and were significantly enriched with an FDR of <10% in at least 2 out of 4 or 6 (GCN5 and PF3D7_1124300) replicates for any of the baits were included. Proteins significant in at least 2 out of 4 replicates for the GFP-control and/or HA-control pulldowns were excluded, as they were likely to represent false-positives. The heatmaps were generated using the web-based Morpheus tool from the Broad Institute (21), plotting the normalized GFP/control or HA/control log2-ratios in a color code (blue-yellow-red) capturing the 0.5 to 99.5 percentile (**Figure 3D** left panel) or 1 to 99 percentile (**Figure 4A**) range of all normalized log2-ratios in the entire dataset file. NaN values - where either a protein is not identified in an experiment or no ratio can be given due to a missing label - are depicted as grey blocks in the heatmap. The right panel in **Figure 3D** represents FDR values for enrichment using a 4 color scale (dark red= very significant binders (FDR 0), bright red= significance cut-off (FDR 0.1), light yellow= sub-significantly recruited proteins (FDR 0.15), blue= non-significant binding (FDR 1), NaN= light grey, with proteins depleted compared to the median normalized PTM-over-unmodified-

control-peptide log2-ratio for each experiment obtaining a FDR of 1). In **Figure 3D**, columns were clustered using a 2-step hierarchical clustering approach; i) baits were clustered on the average log2-norm ratio or average FDR resulting in a very robust order for which the dendrogram is included, ii) hierarchical clustering for the experiments of each particular bait was performed. Rows were ordered manually, first listing the proteins strongly recruited by PHD1 and then ranking based on the strength of recruitment in the combined GCN5 pulldown from high to low summed log2-norm ratios. For **Figure 4A**, hierarchical clustering (HC) was performed on both columns (experiments) and rows (interactors). Repeated clustering of the experiments (columns) yielded (nearly) identical results, therefore the dendrogram is included in the figure. Repeated clustering of the rows was substantially more variable, although the same overall blocks of interactors could mostly be observed. Therefore, no dendrogram for the rows is included and the ranking of interactors in the figure is based on a HC run yielding visually clear separation of relevant groups of interactors. HC was performed using the integrated clustering algorithm of Morphues (22) set at 'one minus Pearson' correlation using the 'average linking' method set at default parameters. In Fig 3D, gene_IDs and protein names (where possible) are included. Red bolded proteins were used for tagging and follow-up in **Figure 3** and, underlined proteins were considered expected chromatin- or transcription-related proteins based on name or domain presence. Proteins that the authors would consider likely contaminants based on protein name, proven localization or domain presence are listed in grey. For **Figure 4A**, baits used for GFP-/HA-IP are color coded in the figure and proteins with (expected) epigenetic or transcription functions based on homology, domain-presence or literature search are underlined. PlasmoDB-based gene names were supplemented with manual curation based on a literature search (references included in figure) and pBLAST searches against 1. *Saccharomyces cerevisiae*, 2. *Homo sapiens* and 3. Non-redundant protein sequences, excluding *Plasmodium* proteins. Previous detection of proteins in cytoplasm and/or nucleus is listed based on Oehring *et al.* (23), where proteins detected by 2 or more peptides in the cytoplasmic or nuclear fraction by Oehring and co-workers or were previously shown to be

cytoplasmic and/or nuclear in literature were marked √, and proteins detected in cytoplasmic or nuclear fraction with only a single peptide were marked ±.

The network-plot of **Figure 4B** was generated in cytoscape (v3.6.1; (24) and further modified in Adobe Illustrator to represents the authors interpretation of BDP-complex composition and connectivity in *P. falciparum*. Nodes represent those proteins from the heatmap in **Figure 4A** that provided solid evidence for being real BDP-complex interactors. Proteins which were only significantly enriched twice (FDR<10%) in all IPs combined (H2A.Z, cdc48, PF3D7_1303800, PF3D7_1419200, PF3D7_1476500) were excluded from the plot, as were proteins of which significance calls were based on single-peptide ratio's only (KLP8) or that were considered to be likely contaminants (ribosomal protein PF3D7_0821700). All nodes were manually positioned in the plot based on evidence from: primarily 1) the number of times an interactor was found to be significantly enriched for any bait , 2) the observed normalized log2-ratio's for all IPs ; and additional evidence from 3) our histone peptide pulldowns, 4) known interactions between orthologous proteins in other organisms, 5) pBLAST searches to identify *S. cerevisiae* or human putative orthologues, 6) publicly available yeast-2-hybrid interaction data (25), 7) information provided on the PlasmoDB gene page (26). Putative BDP-complexes are encircled by dashed lines, resulting in one or more TAF1-(sub)complexes, a BDP1/2-core complex and a BDP4-complex. Edges are indicated for interactions between individual nodes that are within a putative complex (e.g. interaction between SWIB and BDP4, SWIB and PF3D7_0306100, SWIB and PF3D7_1225200) or between non-complex targets and the sum of interactions originating from a putative complex (e.g. interaction between SWIB and the sum of BDP1, BDP2 and PF3D7_1124300). Edge thickness represents the strength of connectivity as measured by the number of times a significantly enriched (FDR<10%) interaction was observed. When only a single significant interaction between two nodes or between a node and a putative complex was observed, the edge has been omitted to remove noise and reduce figure complexity. Interactors were depicted as red ellipses, with baits highlighted in dark green.

*PHD1 SMART and PROMALS3D analysis*

Four PHD domains have been identified in the zinc finger protein PF3D7_1008100 on the PlasmoDB gene page (26)) and were verified by performing an own SMART analysis using default settings (27)). Subsequently. PHD-domain sequences (as listed in PlasmoDB, residues indicated in figure panel) were aligned using a structure based alignment (PROMALS3D, PROfile Multiple Alignment with predicted Local Structure and 3D constraints (28)) with default settings against human CHD4 PHD domain. Key features of PHD domains were highlighted on the alignment based on reference (29): conserved zinc-coordinating residues are labelled grey (dark grey for those binding zinc 1 and light grey for residues involved in binding of zinc 2) ; two core β-strands (β1 and β2) are highlighted green ; regions involved in ligand recognition and selectivity are numbered I-V as in with residues known to be important for H3K4me3 recognition highlighted blue. The residues expected to be responsible for H3K4me2/me3 binding were conserved only in the fourth PHD domain of PHD1 making this the most likely domain responsible for H3K4me2 and me3 peptide recognition.

*Parasite growth assays*

For the flow cytometry growth curves, the *P. falciparum* TAF1/BDP5 knock sideways lines were diluted to 0.1 % parasitemia on day 0 and split into a culture grown in the presence of 250 nM rapalog or in the absence of it (control) for 4 days. Cells from the culture were thoroughly resuspended and 20 µl culture was mixed with 80 µl of pre-mixed RPMI staining medium to obtain a final concentration of 5 µg/ml Hoechst 33342 and 4,5 µg/ml dihydroethidium. Staining was left to proceed for 20 min at room temperature and then 400 µl of ice cold RPMI containing 0,003 % glutaraldehyde was added. The stained cells were used to determine the parasitemia using a LSRII flow cytometer by counting 100,000 events using the FACSDiva software.

To assay developmental progression *TAF1/*BDP5 knock sideways parasites were synchronized with 5% sorbitol *twice* to obtain ring stages *(in an approximately 9h window)*. The culture was split into two dishes. To one 250 nM rapalog was added (to inactivate BDP5) while the other culture served as

control. Giemsa smears were collected at the late ring, early trophozoite and late trophozoite/schizont stage as well in the next cycle when controls had reached the ring stage again.

*RNA-seq library preparation, high throughput sequencing and data analysis.*

*P. falciparum* 3D7 TAF1/BDP5-2xFKBP-GFP-2xFKBP parasites with and without the Lyn-FRB-T2A-mCherry mislocalizer plasmid (2) were synchronized by repeated sorbitol treatments to a ~4h time window. At 14 hours post red blood cell invasion, cultures were split in two and one half of each line was treated with 250 nM rapalog. Cultures were harvested 2, 6 or 12 hours after knock sideways induction, filtered once over a Plasmodipur filter (EurProxima) and resuspended in RLT buffer (Qiagen) with β-mercaptoethanol. Total RNA was isolated using the RNeasy Plus Mini Kit (Qiagen, #74136) with two on-column DNase treatment (Qiagen, #79254). The resulting eluate was treated with TURBO DNase (Ambion, #AM2238) and purified over RNeasy columns with a last on-column DNase treatment. RNA content was quantified using the Qubit RNA HS Assay (Invitrogen, #Q32855). PolyA-modified transcripts were selected from at least 2 µg of total RNA using the Oligotex mRNA Mini kit (Qiagen, #70022) according to the manufacturer's instructions. Total and polyA-selected RNA were snap-frozen and stored at -80˚C until further processing from which point onwards samples were handled together. RNA fragmentation, cDNA synthesis and directional RNA-seq library preparation were performed as described in (8). In short, RNA was hydrolyzed in 150 µl reactions with 40 mM Tris acetate pH 8.2, 100 mM potassium acetate and 30 mM magnesium acetate for 1 minute and 45 seconds at 85 ˚C. Material was precipitated overnight and first-strand cDNA was synthesized using Superscript III with an AT-corrected random N9 primer and 0.2 µ Actinomycin D. During second strand synthesis, dTTPs were replayced by dUTPs to maintain strand information. Resulting cDNA was quantified using the DeNovix dsDNA High Sensitivity Assay (DeNovix) and 20 ng of cDNA was end-repaired, extended with 3' A-overhangs and ligated to Nextflex sequencing adapters (Bio Scientific). Libraries were treated with USER enzyme (NEB, #M5505L) to degrade the dUTP-containing strand and pre-amplified using the KAPA HiFi HotStart ready mix (KAPA Biosystems, #KM2602) and NEXTflex primer mix (Bio Scientific, #514122) with the following program: 98°C for 2 min; 4 cycles of 98°C for

20 sec, 62°C for 3 min; 62°C for 5 min. Libraries were size selected in E-gel size selection gels (Invitrogen) for 300-400 bp and subsequently amplified for another 10 PCR cycles. Libraries were depleted of primer-dimers using Agencourt AMPure XP beads at a library:beads ratio of 1:1. The DNA content of the resulting libraries was quantified using the DeNovix dsDNA High Sensitivity Assay and fragment size distributions were evaluated in a Bioanalyzer High Sensitivity DNA run (Agilent, # 5067-4627). Libraries were sequenced on a NextSeq 500 system (Illumina) for 2x42 bp (TruSeq SR Cluster Kit v2). Read quality was evaluated using FastqQC (v0.11.2) and showed no signs of adapter contamination or considerable reduced base quality. Fastq files were mapped against the *P. falciparum* 3D7 Genome and the Annotated Transcriptome from PlasmoDB v26 using BWA aln and BWA sampe (version 0.7.10-r789). In both cases, read were filtered for a mapping quality of ≥ 30 and uniquely mapping ("XT:A:U") and split by strand. The genome alignments were used to visualize the data in the UCSC genome browser. Transcriptome alignments resulted in 14.1-22.9M mapped, quality filtered reads per library (sense and antisense combined) and were used to generate the count matrix by counting tags per transcript and strand. Only sense counts were used in the follow-up analysis. These were offset by +1 and normalized using the reads-per-million-per-kilobase (RPKM) method. Genes with a minimum 2-fold change in expression (up or down) in the rapalog condition compared to the control with a minimum RPKM of 5 were identified as changed in expression. RPKM expression levels of these differentially expressed genes were converted to proportion over the sum of RPKM values across the different experimental conditions for each genes and plotted in the web-based Morpheus tool from the Broad institute (21). Clustering was performed by k-means clustering using the one minus Pearson correlation into three clusters with a maximum of 1000 iterations. Data from Toenhake *et. al.* (30) was used to plot relative expression levels (proportion over row sums of RPKM values) of these DE genes over the RBC cycle. Data from López-Barragán *et. al.* (31) were obtained from PlasmoDB r45 and quantile normalized (r package preprocessCore version 1.46.0) to plot expression levels in the RBC cycle as well as in gametocyte and ookinete stages. All plots were generated in R using the ggplot2 package (v3.2.1).

## REFERENCES


1. Walliker, D., Quakyi, I.A., Wellems, T.E., McCutchan, T.F., Szarfman, A., London, W.T., Corcoran, L.M., Burkot, T.R. and Carter, R. (1987) Genetic analysis of the human malaria parasite Plasmodium falciparum. *Science*, **236**, 1661-1666.
2. Birnbaum, J., Flemming, S., Reichard, N., Soares, A.B., Mesen-Ramirez, P., Jonscher, E., Bergmann, B. and Spielmann, T. (2017) A genetic system to study Plasmodium falciparum protein function. *Nat Methods*, **14**, 450-456.
3. Prommana, P., Uthaipibull, C., Wongsombat, C., Kamchonwongpaisan, S., Yuthavong, Y., Knuepfer, E., Holder, A.A. and Shaw, P.J. (2013) Inducible knockdown of Plasmodium gene expression using the glmS ribozyme. *PLoS One*, **8**, e73783.
4. Philip, N. and Waters, A.P. (2015) Conditional Degradation of Plasmodium Calcineurin Reveals Functions in Parasite Colonization of both Host and Vector. *Cell Host Microbe*, **18**, 122-131.
5. Fidock, D.A. and Wellems, T.E. (1997) Transformation with human dihydrofolate reductase renders malaria parasites insensitive to WR99210 but does not affect the intrinsic activity of proguanil. *Proc Natl Acad Sci U S A*, **94**, 10931-10936.
6. Moon, R.W., Hall, J., Rangkuti, F., Ho, Y.S., Almond, N., Mitchell, G.H., Pain, A., Holder, A.A. and Blackman, M.J. (2013) Adaptation of the genetically tractable malaria pathogen Plasmodium knowlesi to continuous culture in human erythrocytes. *Proc Natl Acad Sci U S A*, **110**, 531-536.
7. Bartfai, R., Hoeijmakers, W.A., Salcedo-Amaya, A.M., Smits, A.H., Janssen-Megens, E., Kaan, A., Treeck, M., Gilberger, T.W., Francoijs, K.J. and Stunnenberg, H.G. (2010) H2A.Z demarcates intergenic regions of the plasmodium falciparum epigenome that are dynamically marked by H3K9ac and H3K4me3. *PLoS Pathog*, **6**, e1001223.
8. Kensche, P.R., Hoeijmakers, W.A., Toenhake, C.G., Bras, M., Chappell, L., Berriman, M. and Bartfai, R. (2016) The nucleosome landscape of Plasmodium falciparum reveals chromatin architecture and dynamics of regulatory sequences. *Nucleic Acids Res*, **44**, 2110-2124.
9. Bantscheff, M., Hopf, C., Savitski, M.M., Dittmann, A., Grandi, P., Michon, A.M., Schlegl, J., Abraham, Y., Becher, I., Bergamini, G. *et al.* (2011) Chemoproteomics profiling of HDAC inhibitors reveals selective targeting of HDAC complexes. *Nat Biotechnol*, **29**, 255-265.
10. Vermeulen, M. (2012) Identifying chromatin readers using a SILAC-based histone peptide pull-down approach. *Methods Enzymol*, **512**, 137-160.
11. Vermeulen, M., Mulder, K.W., Denissov, S., Pijnappel, W.W., van Schaik, F.M., Varier, R.A., Baltissen, M.P., Stunnenberg, H.G., Mann, M. and Timmers, H.T. (2007) Selective anchoring of TFIID to nucleosomes by trimethylation of histone H3 lysine 4. *Cell*, **131**, 58-69.
12. Hubner, N.C., Nguyen, L.N., Hornig, N.C. and Stunnenberg, H.G. (2015) A quantitative proteomics tool to identify DNA-protein interactions in primary cells or blood. *J Proteome Res*, **14**, 1315-1329.
13. Boersema, P.J., Raijmakers, R., Lemeer, S., Mohammed, S. and Heck, A.J. (2009) Multiplex peptide stable isotope dimethyl labeling for quantitative proteomics. *Nat Protoc*, **4**, 484-494.
14. Rappsilber, J., Mann, M. and Ishihama, Y. (2007) Protocol for micro-purification, enrichment, pre-fractionation and storage of peptides for proteomics using StageTips. *Nat Protoc*, **2**, 1896-1906.
15. Chang, P.Y., Hom, R.A., Musselman, C.A., Zhu, L., Kuo, A., Gozani, O., Kutateladze, T.G. and Cleary, M.L. (2010) Binding of the MLL PHD3 finger to histone H3K4me3 is required for MLL-dependent gene transcription. *J Mol Biol*, **400**, 137-144.
16. Baymaz, H.I., Spruijt, C.G. and Vermeulen, M. (2014) Identifying nuclear protein-protein interactions using GFP affinity purification and SILAC-based quantitative mass spectrometry. *Methods Mol Biol*, **1188**, 207-226.
17. Gruring, C. and Spielmann, T. (2012) Imaging of live malaria blood stage parasites. *Methods in enzymology*, **506**, 81-92.

18. Cox, J. and Mann, M. (2008) MaxQuant enables high peptide identification rates, individualized p.p.b.-range mass accuracies and proteome-wide protein quantification. *Nat Biotechnol*, **26**, 1367-1372.
19. Tyanova, S., Temu, T., Sinitcyn, P., Carlson, A., Hein, M.Y., Geiger, T., Mann, M. and Cox, J. (2016) The Perseus computational platform for comprehensive analysis of (prote)omics data. *Nat Methods*, **13**, 731-740.
20. R_Core_Team. (2017) R: A Language and Environment for Statistical Computing. https://www.R-project.org.
21. Harvard, B.I. (2017) Morpheus, a versatile matrix visualization and analysis software. https://software.broadinstitute.org/morpheus/.
22. Brancucci, N.M.B., Gerdt, J.P., Wang, C., De Niz, M., Philip, N., Adapa, S.R., Zhang, M., Hitz, E., Niederwieser, I., Boltryk, S.D. *et al.* (2017) Lysophosphatidylcholine Regulates Sexual Stage Differentiation in the Human Malaria Parasite Plasmodium falciparum. *Cell*, **171**, 1532-1544 e1515.
23. Oehring, S.C., Woodcroft, B.J., Moes, S., Wetzel, J., Dietz, O., Pulfer, A., Dekiwadia, C., Maeser, P., Flueck, C., Witmer, K. *et al.* (2012) Organellar proteomics reveals hundreds of novel nuclear proteins in the malaria parasite Plasmodium falciparum. *Genome Biol*, **13**, R108.
24. Shannon, P., Markiel, A., Ozier, O., Baliga, N.S., Wang, J.T., Ramage, D., Amin, N., Schwikowski, B. and Ideker, T. (2003) Cytoscape: a software environment for integrated models of biomolecular interaction networks. *Genome Res*, **13**, 2498-2504.
25. LaCount, D.J., Vignali, M., Chettier, R., Phansalkar, A., Bell, R., Hesselberth, J.R., Schoenfeld, L.W., Ota, I., Sahasrabudhe, S., Kurschner, C. *et al.* (2005) A protein interaction network of the malaria parasite Plasmodium falciparum. *Nature*, **438**, 103-107.
26. Aurrecoechea, C., Brestelli, J., Brunk, B.P., Fischer, S., Gajria, B., Gao, X., Gingle, A., Grant, G., Harb, O.S., Heiges, M. *et al.* (2010) EuPathDB: a portal to eukaryotic pathogen databases. *Nucleic Acids Res*, **38**, D415-419.
27. Schultz, J., Milpetz, F., Bork, P. and Ponting, C.P. (1998) SMART, a simple modular architecture research tool: identification of signaling domains. *Proc Natl Acad Sci U S A*, **95**, 5857-5864.
28. Pei, J., Kim, B.H. and Grishin, N.V. (2008) PROMALS3D: a tool for multiple protein sequence and structure alignments. *Nucleic Acids Res*, **36**, 2295-2300.
29. Sanchez, R. and Zhou, M.M. (2011) The PHD finger: a versatile epigenome reader. *Trends Biochem Sci*, **36**, 364-372.
30. Toenhake, C.G., Fraschka, S.A., Vijayabaskar, M.S., Westhead, D.R., van Heeringen, S.J. and Bartfai, R. (2018) Chromatin Accessibility-Based Characterization of the Gene Regulatory Network Underlying Plasmodium falciparum Blood-Stage Development. *Cell Host Microbe*, **23**, 557-569 e559.
31. Lopez-Barragan, M.J., Lemieux, J., Quinones, M., Williamson, K.C., Molina-Cruz, A., Cui, K., Barillas-Mury, C., Zhao, K. and Su, X.Z. (2011) Directional gene expression and antisense transcripts in sexual and asexual stages of Plasmodium falciparum. *BMC Genomics*, **12**, 587.
